# Proteome Modulation in Peripheral Blood Mononuclear Cells of *Peste des Petits* Ruminants Vaccinated Goats and Sheep

**DOI:** 10.3389/fvets.2021.670968

**Published:** 2021-09-22

**Authors:** Sajad Ahmad Wani, Amit Ranjan Sahu, Raja Ishaq Nabi Khan, Manas Ranjan Praharaj, Shikha Saxena, Kaushal Kishor Rajak, Dhanavelu Muthuchelvan, Aditya Sahoo, Bina Mishra, R. K. Singh, Bishnu Prasad Mishra, Ravi Kumar Gandham

**Affiliations:** ^1^Division of Veterinary Biotechnology, Indian Council of Agricultural Research - Indian Veterinary Research Institute, Bareilly, India; ^2^College of Pharmacy, Pharmaceutics and Pharmaceutical Chemistry, The Ohio State University, Columbus, OH, United States; ^3^Systems Biology Lab, Department of Biotechnology -National Institute of Animal Biotechnology, Hyderabad, India; ^4^Division of Biological Products, Indian Council of Agricultural Research - Indian Veterinary Research Institute, Bareilly, India; ^5^Division of Virology, Indian Council of Agricultural Research - Indian Veterinary Research Institute, Mukteswar, India

**Keywords:** Sungri/96, PPRV, proteome, immunity, vaccine

## Abstract

In the present study, healthy goats and sheep (*n* = 5) that were confirmed negative for *peste des petits* ruminants virus (PPRV) antibodies by monoclonal antibody-based competitive ELISA and by serum neutralization test and for PPRV antigen by s-ELISA were vaccinated with Sungri/96. A quantitative study was carried out to compare the proteome of peripheral blood mononuclear cells (PBMCs) of vaccinated goat and sheep [5 days post-vaccination (dpv) and 14 dpv] vs. unvaccinated (0 day) to divulge the alteration in protein expression following vaccination. A total of 232 and 915 proteins were differentially expressed at 5 and 14 dpv, respectively, in goats. Similarly, 167 and 207 proteins were differentially expressed at 5 and 14 dpv, respectively, in sheep. Network generated by Ingenuity Pathway Analysis was “infectious diseases, antimicrobial response, and inflammatory response,” which includes the highest number of focus molecules. The bio functions, cell-mediated immune response, and humoral immune response were highly enriched in goats at 5 dpv and at 14 dpv. At the molecular level, the immune response produced by the PPRV vaccine virus in goats is effectively coordinated and stronger than that in sheep, though the vaccine provides protection from virulent virus challenge in both. The altered expression of certain PBMC proteins especially ISG15 and IRF7 induces marked changes in cellular signaling pathways to coordinate host immune responses.

## Introduction

*Peste des petits* ruminants (PPR) is a severe, contagious viral disease of small ruminants, mainly sheep and goats, caused by *peste des petits* ruminants virus (PPRV) belonging to the genus *Morbillivirus* and family *Paramyxoviridae* (1). The disease is endemic in many countries of Asia, the Middle East, and Africa (1). The disease is clinically manifested by fever, anorexia, ocular and nasal discharge, erosions and ulcers in the digestive mucosa, diarrhea, and marked leukopenia with immunosuppression and may lead to death (2). The disease is classified as a World Organisation for Animal Health (OIE)-listed disease. The disease causes severe economic losses, as mortality and morbidity can reach 90–100% (3).

Live attenuated vaccines Nigeria 75/1 and Sungri/96 have been widely used for the control of PPRV in Africa (4) and India (5), respectively. Sungri/96 vaccine is believed to provide protective immunity in sheep and goats for years (3). This immune response that results in the protection of hosts after vaccination is attributed to innate and both humoral and cell-mediated immunity, which, however, warrants further investigation (6–11).

It is well-known that peripheral blood mononuclear cells (PBMCs) play a vital role in the immune response (12) and have been widely used in an *in vitro* model to study host–PPRV interactions and in other morbillivirus infections (9, 10, 13, 14). Transcriptome profiling has uncovered transcription factors and miRNAs in modulating the immune response to PPRV Sungri/96 live attenuated vaccine strain *in vitro* in PBMCs (9) and virulent PPRV infection (15, 16). To date, there are no *in vivo* or *in vitro* reports of proteomics profiling of PBMCs in PPRV-vaccinated goats and sheep.

Vaccination of sheep and goats with live attenuated virus is shown to elicit protective immunity to infection with virulent PPRV; yet, the mechanisms that induce immune response and confer protection from virulent PPRV strains are still not completely clear. In this study, we examine the proteome changes that occur in PBMCs of sheep and goat in response to PPRV vaccination, which contribute to the development of immunity, by identifying differentially expressed proteins (DEPs) and biological pathways associated at the early time points. To establish differences in vaccine response in sheep and goat, DEPs in PBMCs at 5 and 14 dpv in comparison to 0 dpv as control were identified. Thereafter, we performed extensive pathway and network analyses to find out differences in the underlying protein pathways associated with PBMCs of sheep and goat at different time points.

## Materials and Methods

### Animal Experiment, Ethics Statement, and Virus

In the present study, healthy goats (*n* = 5) and sheep (*n* = 5) confirmed negative for PPRV antibodies by monoclonal antibody-based competitive ELISA (17) and by serum neutralization test (SNT) (18) and for PPRV antigen by s-ELISA (17) were used. PPRV live attenuated vaccine virus (Sungri/96) was used in this experiment to vaccinate sheep and goats. The study was carried out after obtaining permission from the Indian Veterinary Research Institute Animal Ethics Committee (IVRI-IAEC) under the Committee for the Purpose of Control and Supervision of Experiments on Animals (CPCSEA), India.

### Study Design

Goats and sheep (each *n* = 5) were vaccinated, and PBMCs were isolated from the animals before (0 day) and 5 and 14 days post-vaccination (5 and 14 dpv, respectively). Thereafter, proteome profiling of PBMCs isolated at the different time points, 0 day, 5 dpv, and 14 dpv, was carried out. DEPs at 5 and 14 dpv were identified, respectively, by comparing the proteome data of 5- and 14-dpv PBMCs with 0-day PBMC data.

### Serological Response to Vaccination/Infection

Detection of PPRV antibodies in the sera samples collected from PPRV-vaccinated animals was carried out using monoclonal antibody (directed to H protein)-based competitive-ELISA test (17). The samples that had a percent inhibition (PI) value of >40% were considered positive.

### Isolation of Peripheral Blood Mononuclear Cells and Protein Isolation

The PBMC subset isolation was carried out following the standard procedure as described in our previous study (16). For protein isolation, ~10^7^ cells of PBMCs were taken in lysis buffer [50 mM Tris Buffer + protease inhibitor cocktail (PIC) + phenylmethylsulfonyl fluoride (PMSF)], homogenized on ice, centrifuged at 14,000 rpm, and the supernatant was collected into a separate tube (Tris Buffer extract). Urea lysis buffer was added to the cell pellet. This was followed by centrifugation at 14,000 rpm and collection of the resultant supernatant. For quality check, the supernatant was run on a sodium dodecyl sulfate-polyacrylamide gel electrophoresis (SDS-PAGE). Protein concentration was determined using the Bradford assay, and 100 μg of the samples was taken for digestion.

### Sample Preparation for Liquid Chromatography–Mass Spectrometry Analysis

The samples were added with 100 mM dithiothreitol (DTT) for 1 h at 95°C, treated with 250 mM iminodiacetic acid (IDA), and kept for 45 min at room temperature in the dark. This was followed by digestion of the sample with trypsin and overnight incubation at 37°C. Thereafter, formic acid (1%) was added to it, incubated at 37°C for 1 h and vacuum dried. The resulting sample was dissolved in 10 μl of 0.1% formic acid and centrifuged at 10,000 × g. The supernatant was injected on C18 Nano-LC column for separation of peptides. The Waters Synapt G2 Q-TOF instrument for MS and MS/MS were used for analysis.

### Liquid Chromatography–Mass Spectrometry Analysis

Through MassLynx 4.1 Waters, the raw data were processed, and through PLGS software, the proteins were identified. The threshold while considering a protein was as follows: Minimum number of peptides to be found for a protein, 2; Minimum number of fragment (MSMS) ions in a peptide, 3; Minimum number of fragment (MSMS) ions in a protein, 7; Peptide mass tolerance, 30 ppm; and Fragment ion mass tolerance, 70 ppm. The quantification of the protein was carried out through Expression analysis package of the PLGS software, and the identified proteins in the three runs of each sample were compared with each other as control (healthy) and vaccinated samples.

### Ingenuity Pathway Analysis

Data were analyzed using QIAGEN's IPA (QIAGEN, Redwood City, USA). The uniport ID of DEPs was converted into gene ID from uniport data set, and this list of genes with corresponding fold change was uploaded to IPA for downstream analysis. The canonical pathways and functional processes of biological importance that were the most significant were identified using the list of DEPs identified with liquid chromatography–mass spectrometry (LC-MS) and the Ingenuity Pathways Knowledge Base (IKB). Pathway enrichment *p*-values (Fisher's exact test) and activation z-scores were calculated by IPA. Core analysis and comparison analysis for each dataset were performed to know significant (*p* < 0.05) and activated (Z score > 2) or inactivated (Z score < −2) canonical pathways. Network analysis was also performed.

## Results

In this study, sheep and goats screened -ve for PPRV antibodies by competitive-ELISA and SNT and for PPRV antigen by s-ELISA were used. Vaccinated goats were found seropositive by 9 dpv, while sheep had an idiosyncratic response in the range of 9–14 dpv for different animals (19).

### Proteomics Profiling of Peripheral Blood Mononuclear Cells of Vaccinated Goats and Sheep Results in Identification of Unique and Common Differentially Expressed Proteins

**Figure 1:**
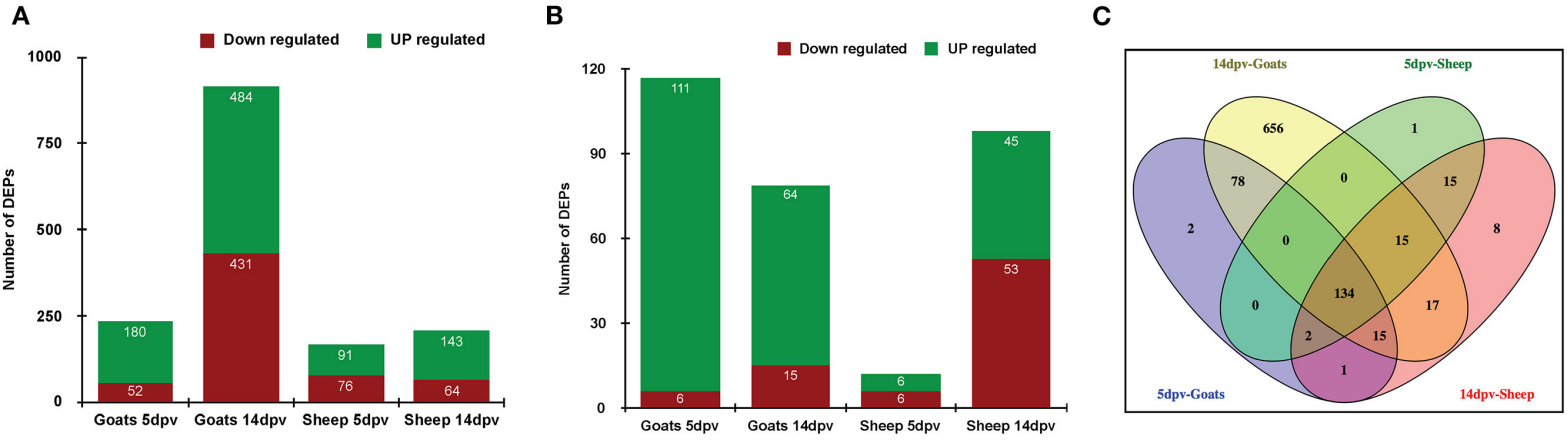
**(A)** Differentially expressed proteins (DEPs) in vaccinated goats and sheep at 5 days post-vaccination (dpv) and 14 dpv. **(B)** DEPs in vaccinated goats and sheep at 5 and 14 dpv with cutoff fold change >2 for upregulated and <0.5 for downregulated proteins. **(C)** Venn diagram representing common and unique DEPs between sheep and goats at 5 and 14 dpv.

A quantitative study was carried out to compare the proteome of PBMCs of vaccinated goat and sheep (5 and 14 dpv) vs. unvaccinated (0 day). A total of 232 and 915 proteins were differentially expressed at 5 and 14 dpv, respectively, in goats. Similarly, 167 and 207 proteins were differentially expressed at 5 and 14 dpv, respectively, in sheep (Figure 1A) at a cutoff fold change >2 for upregulated and <0.5 for downregulated proteins. The number of upregulated DEPs in goats at 5 and 14 dpv was 111 and 64, respectively, whereas this was 6 and 45 in sheep, respectively (Figure 1B). A higher number of significantly upregulated proteins were found in goats at 5 dpv. A total number of 134 DEPs were found common among “5dpv-Goats,” “14dpv-Goats,” “5dpv-Sheep,” and “14dpv-Sheep” (Figure 1C). FGG, SOCS7, TNFSF10, and CD274 were the most upregulated DEPs at 5 and 14 dpv in goat and sheep, respectively.

### Protein Set Enrichment Analysis Using the Cytoscape Plugin ClueGO Identified Gene Ontology Terms Enriched in Peripheral Blood Mononuclear Cells of Vaccinated Goats and Sheep

**Figure 2:**
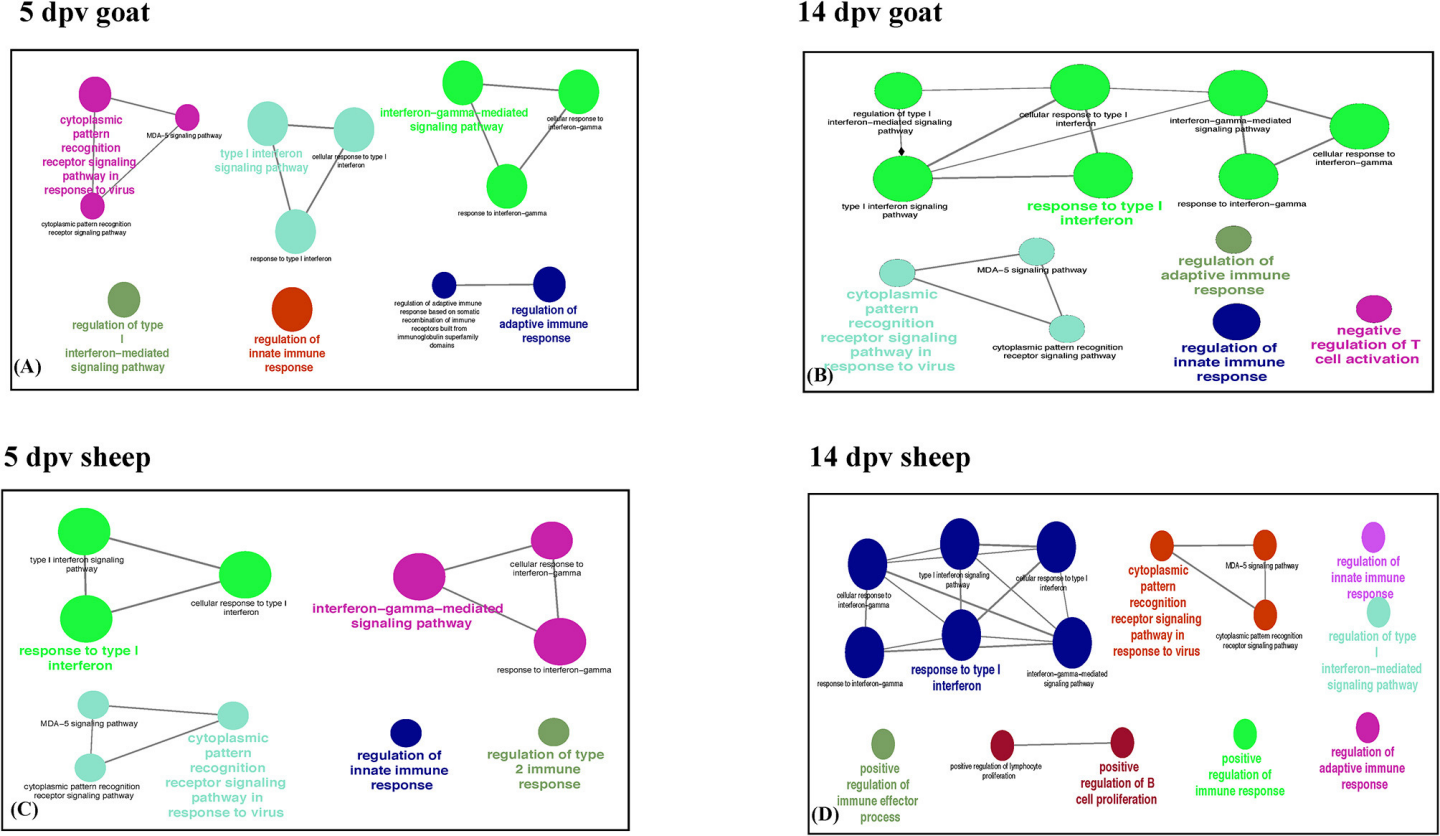
Protein set enrichment analysis using the ClueGO at **(A)** 5 days post-vaccination (dpv) and **(B)** 14 dpv vaccinated goats and **(C)** 5 dpv and **(D)** 14 dpv vaccinated sheep. The edges of the resulting ClueGO network are based on kappa statistics and reflect the relationships between the GO terms (network nodes) based on the similarity of their associated proteins. The proteins associated with significant GO categories are displayed as well as the *p*-value associated with the GO term. In the network, only significantly enriched categories (*p*-value 0.05, Bonferroni corrected) are shown. The size of the node indicates significance, and the color indicates a group.

Immune system processes enriched in goats at 5 dpv were type I interferon signaling, cytoplasmic PRR signaling pathway in response to virus, interferon gamma-mediated signaling pathway, regulation of innate immune response, and regulation of adaptive immune response (Figure 2A), whereas at 14 dpv, response to type I interferon, type I interferon signaling, interferon gamma-mediated signaling pathway, cytoplasmic PRR signaling pathway in response to virus, regulation of innate immune response, negative regulation of T-cell activation, and regulation of adaptive immune response were found enriched (Figure 2B). In vaccinated sheep at 5 dpv, interferon gamma-mediated signaling pathway, response to type I interferon, cytoplasmic PRR signaling pathway in response to virus, regulation of innate immune response, and regulation of type 2 immune response were enriched (Figure 2C). At 14 dpv in sheep, response to type I interferon, interferon gamma-mediated signaling pathway, cytoplasmic PRR signaling pathway in response to virus, regulation of innate immune response, regulation of adaptive immune response, and positive regulation of B-cell proliferation were enriched (Figure 2D).

### Ingenuity Pathway Analysis Reveals Canonical Pathways That Were Perturbed in Peripheral Blood Mononuclear Cells of Vaccinated Goats and Sheep

**Figure 3:**
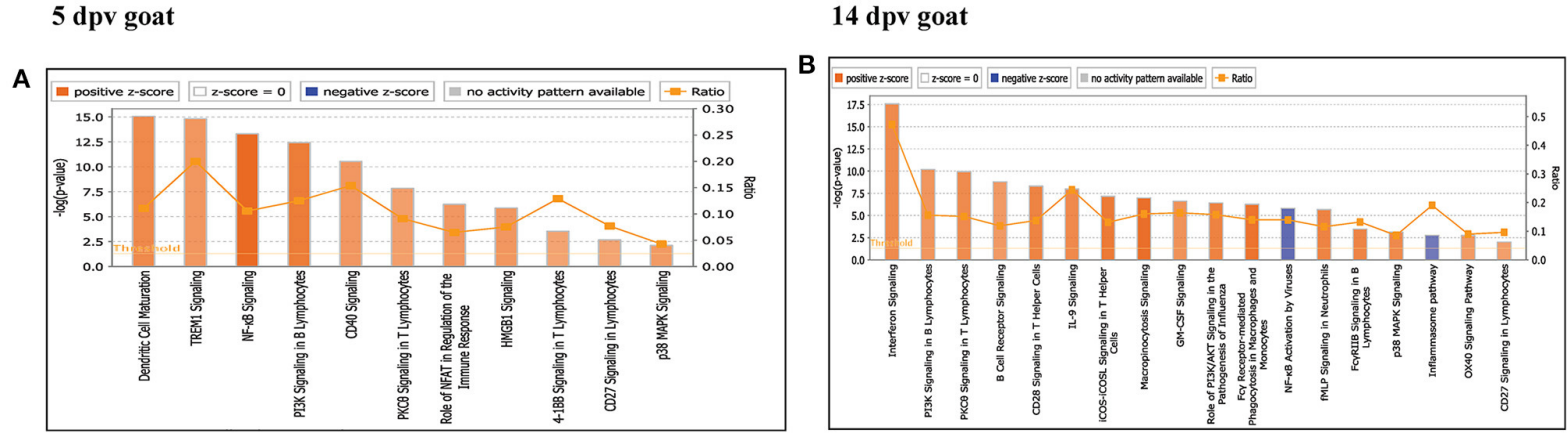
Canonical pathways activated/inactivated in vaccinated goats **(A)** 5 days post-vaccination (dpv) and **(B)** 14 dpv were generated in core analysis of the Ingenuity Pathway Analysis (IPA) tool. Red pathways are activated (>2), and blue pathways are inactivated (<2). Height of the bar graphs indicates -log (*p*-value), and the line graph shows the ratio of listed proteins found in each pathway over the total number of proteins in that pathway. The uniport ID of differentially expressed proteins (DEPs) was converted into gene ID, and this list of proteins with corresponding fold change was uploaded in IPA for downstream analysis.

**Figure 4:**
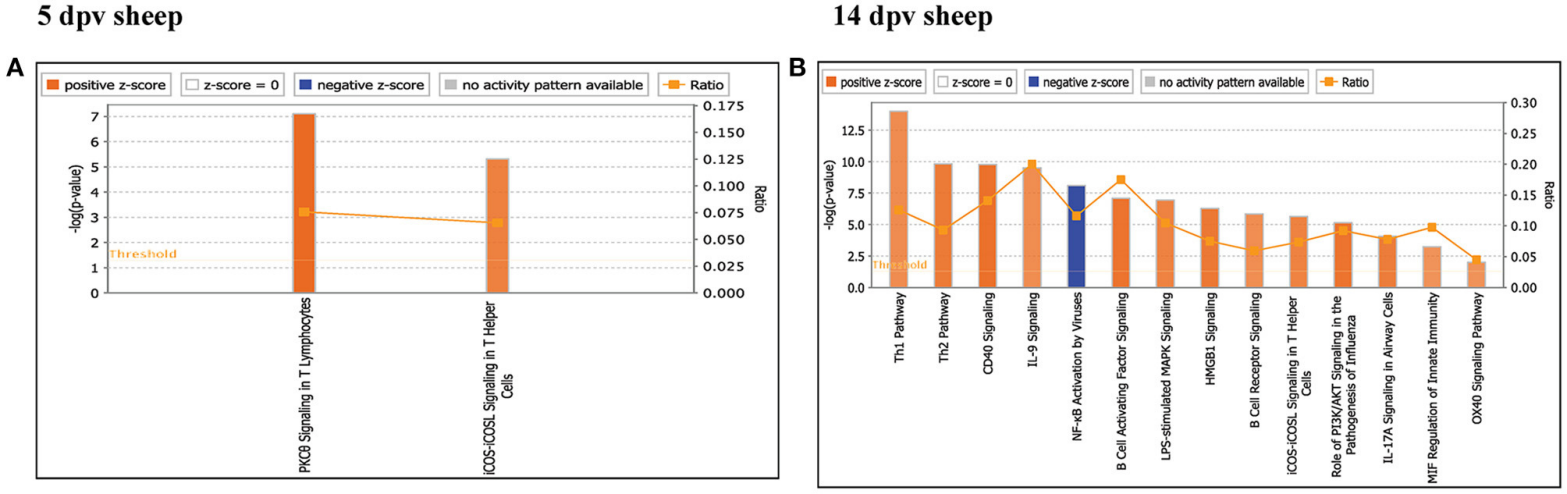
Canonical pathways activated/inactivated in vaccinated sheep **(A)** 5 days post-vaccination (dpv) and **(B)** 14 dpv were generated in core analysis of the Ingenuity Pathway Analysis (IPA) tool. Red pathways are activated (>2), and blue pathways are inactivated (<2). Height of the bar graphs indicates -log (*p*-value), and the line graph shows the ratio of listed proteins found in each pathway over the total number of proteins in that pathway. The uniport ID of differentially expressed proteins (DEPs) was converted into gene ID, and this list of proteins with corresponding fold change was uploaded in IPA for downstream analysis.

Core analysis for each dataset was performed to know significant (*p* < 0.05) and activated (Z score > 2) or inactivated (Z score < −2) canonical pathways. In vaccinated goats at 5 dpv, all the pathways were found to be activated (Z score > 2) like dendritic cell maturation, TREM1 signaling, nuclear factor (NF)-κB signaling, phosphoinositide 3-kinase (PI3K) signaling in B lymphocytes, etc. Dendritic cell maturation pathway was the most significantly enriched (–log *p*-value) pathway. The ratio—number of the proteins in the dataset mapping to each specific pathway divided by the total number of proteins in the pathway—was highest for TREM1 pathway (Figure 3A). In vaccinated goats at 14 dpv, all the pathways, e.g., interferon signaling, PI3K signaling in B lymphocytes, protein kinase C (PKC) theta signaling in T lymphocytes, B-cell receptor signaling, CD28 signaling in T helper cells, etc., were activated except the nuclear factor (NF)-κB activation by viruses and inflammasome pathway. Interferon signaling was the most significantly enriched (–log *p*-value) pathway with highest ratio also (Figure 3B). In vaccinated sheep at 5 dpv, PKC theta signaling in T lymphocytes and inducible costimulator (ICOS)–inducible costimulator ligand (ICOSL) signaling in T helper cells were activated. PKC theta signaling in T lymphocytes was having the highest ratio with significant enrichment (Figure 4A). In vaccinated sheep at 14 dpv, all the pathways, e.g., Th1 pathway, Th2 pathway, CD40 signaling, interleukin (IL)-9 signaling pathway, etc., were activated except the NF-κB activation by viruses. Th1 pathway was the most significantly enriched (–log *p*-value) pathway, and IL-9 signaling pathway had the highest ratio (Figure 4B).

### Annotation of Differentially Expressed Proteins in Peripheral Blood Mononuclear Cells of Vaccinated Goats and Sheep

The DEPs in vaccinated goats and sheep at 5 and 14 dpv have been characterized with specific bio functions in IPA. DEPs of vaccinated goats at 5 dpv were distributed into 84 bio functions activated (Z score > 2) or inactivated (Z score < −2) (Supplementary Table 1). Stimulation of cells, phosphorylation of protein, and transcription of RNA were the top 3 activated bio functions, and the number of proteins involved in each bio function was 25, 39, and 80, respectively. DEPs of vaccinated goats at 14 dpv were distributed into 48 bio functions activated (Z score > 2) or inactivated (Z score < −2) (Supplementary Table 2). Apoptosis of epithelial cells, function of mononuclear leukocytes, immune response of cells, and function of lymphatic system cells were the top 4 activated bio functions, and the number of proteins involved in each bio function was 61, 59, 114, and 62, respectively. DEPs of vaccinated sheep at 5 dpv were distributed into 22 bio functions activated (Z score > 2) or inactivated (Z score < −2) (Supplementary Table 3). Quantity of IgG, quantity of immunoglobulin, quantity of double-positive thymocyte, and production of protein were the top 4 activated bio functions, and the number of proteins involved in each bio function was 19, 28, 10, and 32, respectively. DEPs of vaccinated sheep at 14 dpv were distributed into 84 bio functions activated (Z score > 2) or inactivated (Z score < −2) (Supplementary Table 4). Quantity of leukocytes, quantity of lymphatic system cells, quantity of mononuclear leukocytes, and proliferation of B lymphocytes were the top 4 activated bio functions, and the number of proteins involved in each bio function was 76, 67, 65, and 34, respectively.

### Network Analysis Identified the Key Networks in Peripheral Blood Mononuclear Cells of Vaccinated Goats and Sheep

**Figure 5:**
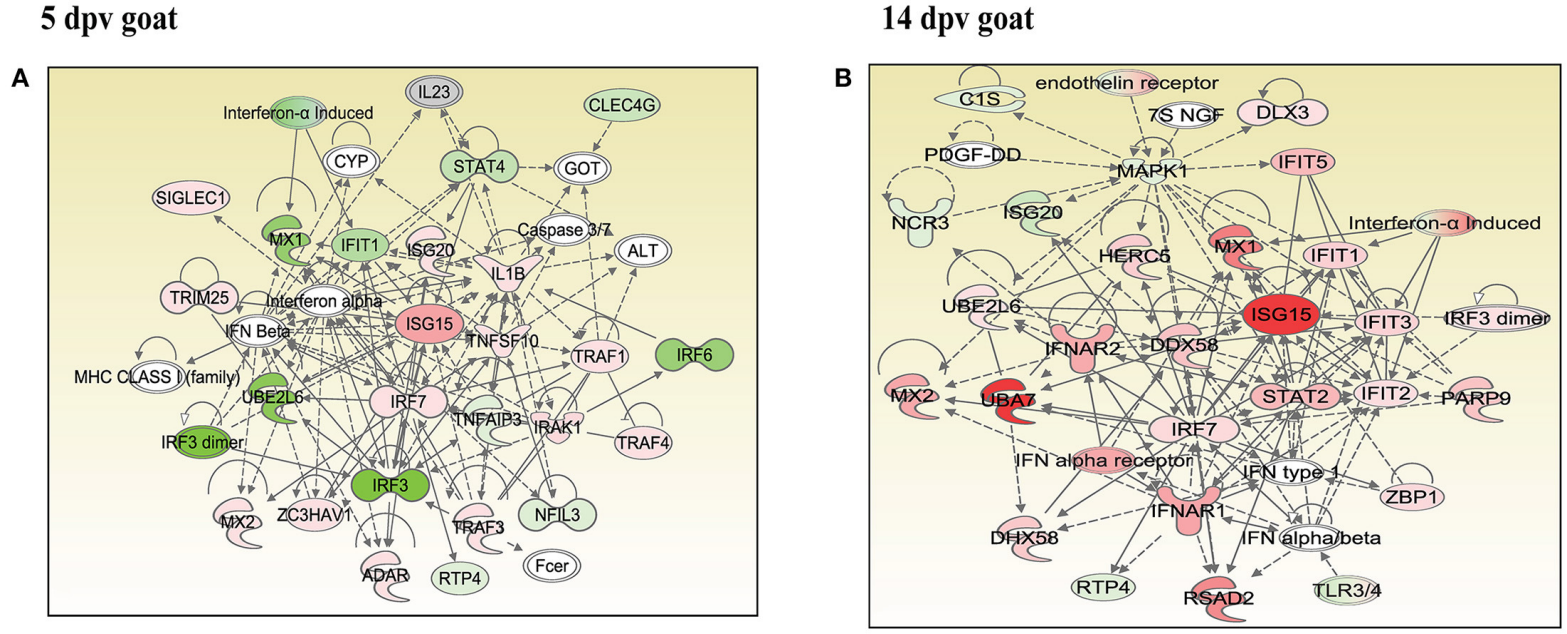
Network generated in Ingenuity Pathway Analysis tool of “infectious diseases, antimicrobial response, and inflammatory response” of differentially expressed proteins (DEPs) in vaccinated goats at **(A)** 5 days post-vaccination (dpv) and **(B)** 14 dpv. Proteins that were upregulated are shown in red, and those that were downregulated are shown in green. Proteins in white were not significantly dysregulated. Symbol shape indicates gene function.

**Figure 6:**
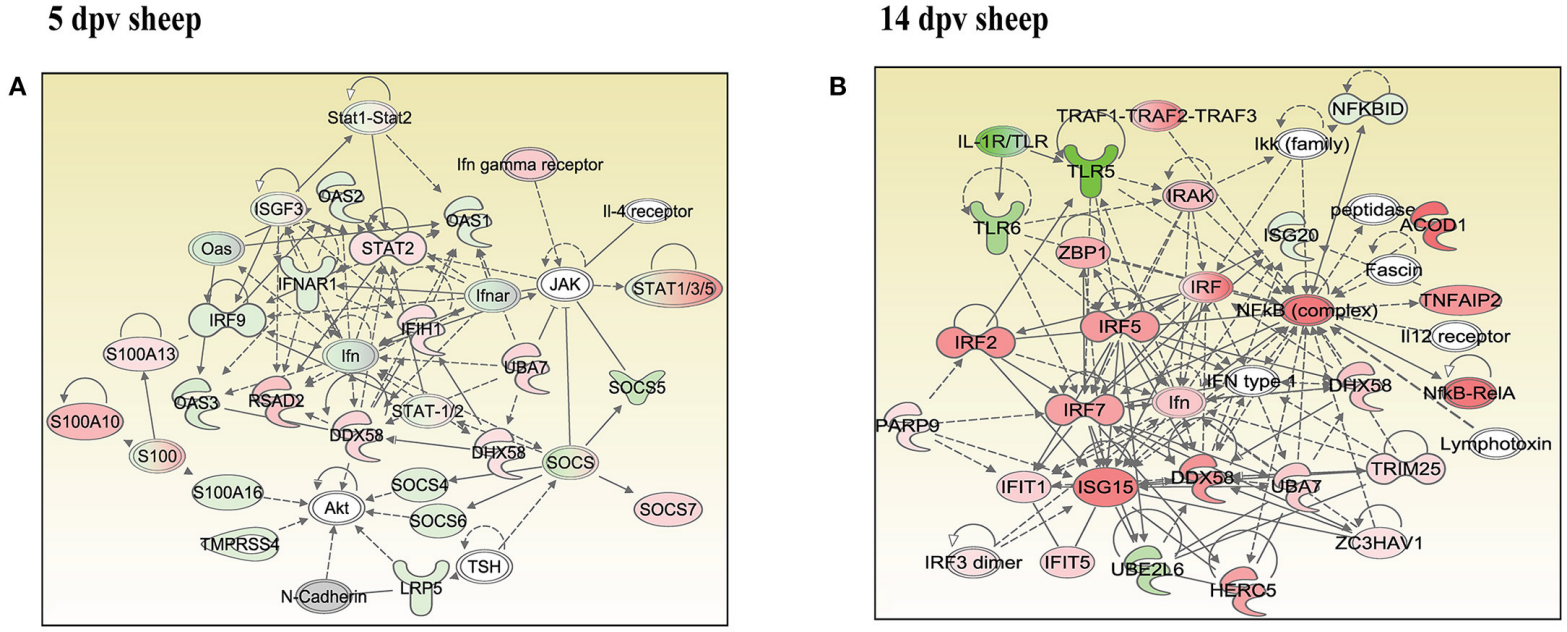
Network generated in Ingenuity Pathway Analysis tool of “infectious diseases, antimicrobial response, and inflammatory response” of differentially expressed proteins (DEPs) in vaccinated sheep at **(A)** 5 days post-vaccination (dpv) and **(B)** 14 dpv. Proteins that were upregulated are shown in red, and those that were downregulated are shown in green. Proteins in white were not significantly dysregulated. Symbol shape indicates gene function.

In vaccinated goats at 5 dpv, 19 networks were generated, out of which networks 3, 9, 11, and 15 were overlapping (Supplementary Table 5). The network with the highest score (43) was “infectious diseases, antimicrobial response, and inflammatory response,” with 24 focus molecules (Figure 5A). The key proteins upregulated in the highest scoring network were ISG15, IRF7, IL1B, TNFSF10, etc. In vaccinated goats at 14 dpv, 25 networks were generated, out of which networks 2, 4, 6, 9, 13, 23, and 24 were overlapping (Supplementary Table 6). The network with the highest score (40) was “developmental disorder, hereditary disorder, metabolic disease,” with 27 focus molecules (Figure 5B). Furthermore, “infectious diseases, antimicrobial response, and inflammatory response” was the second network with scores of 36 and 25 focus molecules. The key proteins upregulated in the highest scoring network were TMPRSS2, MEF2C, HES1, ACTC1, etc., and the key proteins upregulated in the second highest scoring network were ISG15, UBA7, MX1, RSAD2, IFIT3, etc. In vaccinated sheep at 5 dpv, 14 networks were generated, out of which networks 5, 7, and 9 were overlapping (Supplementary Table 7). The network with the highest score (20) was “antimicrobial response, inflammatory response, infectious diseases,” with 20 focus molecules (Figure 6A). The key proteins upregulated in the highest scoring network were S100A10, RSAD2, DDX58, IFIH1, etc. In vaccinated sheep at 14 dpv, 18 networks were generated, out of which networks 6, 9, and 10 were overlapping (Supplementary Table 8). The network with the highest score (21) was “antimicrobial response, inflammatory response, infectious diseases,” with 21 focus molecules (Figure 6B). The key proteins upregulated in the highest scoring network were ISG15, IRF7, NFKB complex, TNFAIP2, HERC5, ACOD1, etc.

### Comparison Analysis of Canonical Pathways Between Peripheral Blood Mononuclear Cells of Vaccinated Goats and Sheep

**Figure 7:**
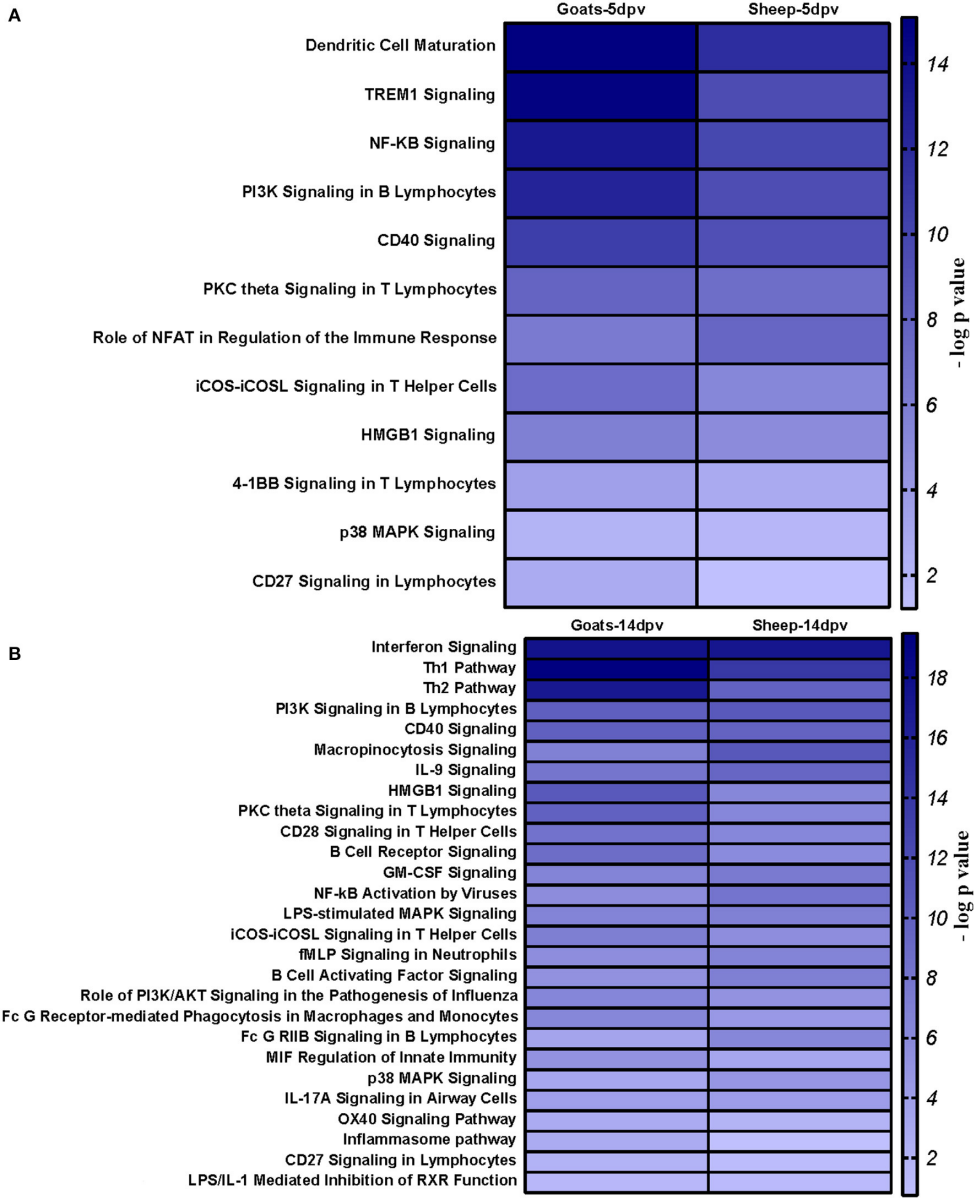
Comparison analysis of canonical pathways significantly enriched in vaccinated goats and sheep at **(A)** 5 days post-vaccination (dpv) and **(B)** 14 dpv generated in the Ingenuity Pathway Analysis tool.

**Figure 8:**
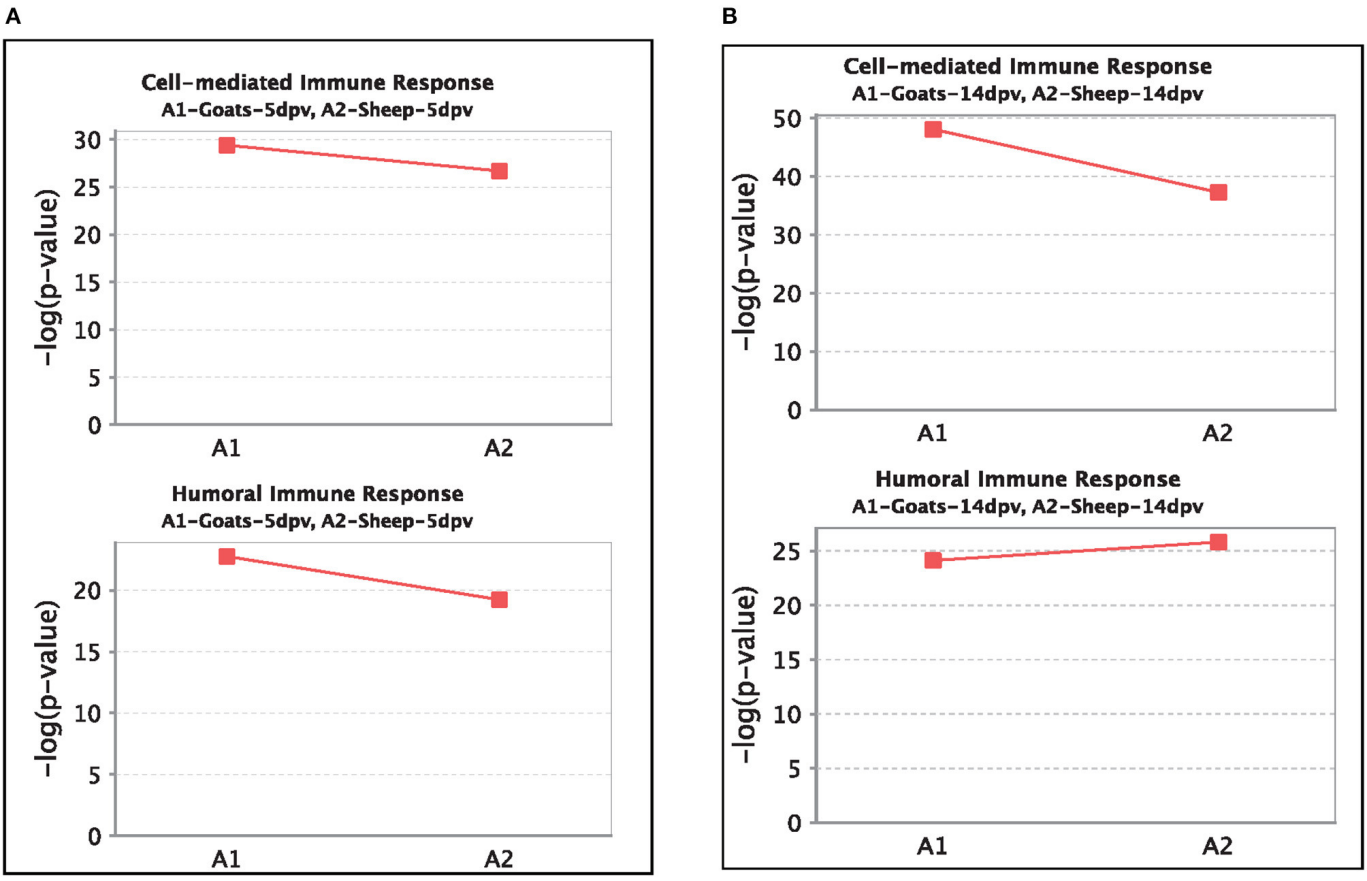
Comparison of significant enrichment (–log *p*-value) of proteins in cell-mediated immune response and humoral immune response bio functions between vaccinated sheep and goats at **(A)** 5 days post-vaccination (dpv) and **(B)** 14 dpv.

At 5 dpv, most of the immune-related pathways were found to be more significantly (–log *p*-value) enriched in goats than in sheep. Dendritic cell maturation, TREM1, NF-κB signaling, icos-icosL signaling in T helper cells, PI3K signaling in B lymphocytes, CD27 signaling in lymphocytes, etc., showed higher enrichment in goats, whereas in sheep, the role of nuclear factor of activated T cells (NFAT) in the regulation of the immune response was highly enriched (Figure 7A). At 14 dpv, Th1 pathway, Th2 pathway, high-mobility group box protein 1 (HMGB1) signaling, Fc gamma receptor-mediated phagocytosis, macrophage migration inhibitory factor (MIF) regulation of innate immunity, PKC theta signaling, B-cell receptor signaling, CD28 signaling T helper cells, inflammasone pathway, and CD27 signaling in lymphocytes were significantly enrichment in goats. In sheep, granulocyte-macrophage colony-stimulating factor (GM-CSF) signaling, NF-κB signaling by viruses, B-cell activating factor signaling, Fc gamma R II signaling in B lymphocytes, and p38 mitogen-activated protein kinase (MAPK) signaling were enriched in sheep (Figure 7B). The bio functions cell-mediated immune response and humoral immune response were highly enriched in goats at 5 dpv (Figure 8A). At 14 dpv, cell-mediated immune response was highly enriched in goats, with humoral immune response being equally enriched (Figure 8B).

## Discussion

Proteomics facilitates the identification of system-wide changes in protein expression (22, 23) and in combination with bioinformatics analyses has been used to study the host–pathogen interaction (24). Since changes in protein expression in PBMCs of vaccinated goats and sheep with PPRV were unknown, LC-MS was applied in this study.

A quantitative study carried out to compare the proteome of PBMCs of vaccinated goat and sheep (5 and 14 dpv) vs. control (0 dpv) revealed enrichment of immune system processes and orchestration of antiviral state by type I interferon signaling, cytoplasmic PRR signaling pathway in response to virus, interferon gamma-mediated signaling pathway, regulation of innate immune response, regulation of adaptive immune response, negative regulation of T-cell activation, regulation of type 2 immune response, positive regulation of B-cell proliferation. From transcriptome analysis of PBMCs in *in vitro* studies by RNA sequencing, PPRV is reported to create a strong antiviral state by enrichment of Toll-like receptor (TLR) signaling pathways, innate immune response, inflammatory response, positive regulation of signal transduction and cytokine production, RIG signaling pathway, chemokine signaling pathway, Class-I major histocompatibility complex (MHC) presentation and processing, and classical antibody-mediated complement activation (9, 10, 25). Initially, the PPR vaccine virus induces an innate immune response, which further shapes a strong adaptive immune response.

“Infectious diseases, antimicrobial response, and inflammatory response” was the network generated by IPA with highest score at 5 dpv vaccinated goats, with the second highest score at 14 dpv vaccinated goats, and highest score at 5 and 14 dpv vaccinated sheep. The key proteins identified in network analysis were ISG15, IRF7, IL1B, and TNFSF10 at 5 dpv and ISG15, UBA7, MX1, RSAD2, IFIT3, and TMPRSS2 at 14 dpv in goats. At the transcriptome level, the high expression levels of viral sensors MDA5 (IFIH1), R1G-1 (DDX58), and ISGS (*ISG15, Mx1, Mx2, RSAD2, IFIT3*, and *IFIT5*) and transcription factors like IRF-7 and STAT-1 that induce ISG expression were found in lymphocytes during virulent PPRV infection (25). In sheep, S100A10, RSAD2, DDX58, and IFIH1 were the key proteins at 5 dpv, and ISG15, IRF7, NFKB complex, TNFAIP2, HERC5, and ACOD1 were the key proteins at 14 dpv. ISG15, IRF7, MX1, RSAD2, and IFIT3 have been found to play a vital role in modulating immune response against the viral infection (26–33). The upregulation of genes of the key immune molecules like ISG15 in all cell subsets and IFIT3, MX1, and ISG15 in certain cell subsets of the sheep PBMCs was found at earlier stages (5 dpv) of PPRV vaccination (11). In goats, the elevated levels of the genes of ISG20, ISG15, IRF7, various IFITs, MX1, and MX2 was found at 5 dpv (11). The altered expression of these PBMC proteins, identified following PPRV vaccination of sheep and goats, suggests marked changes in cellular signaling pathways to modulate host responses.

At 5 dpv, most of the immune-related pathways were found to be more significantly (–log *p*-value) enriched in goats than in sheep. Dendritic cell maturation, TREM1 signaling, NF-κB signaling, ICOS-ICOSL signaling in T helper cells, PI3K signaling in B lymphocytes, CD27 signaling in lymphocytes, etc., showed higher enrichment in goats. TREM1 regulates T-cell proliferation and activates antigen-presenting cells (34, 35). NF-κB signaling induces transcription of cytokines and antivirals (36). ICOSL-mediated signaling, PI3K signaling, and CD27 signaling stimulate T- and B-cell differentiation and maturation (20, 21, 37–39). The bio functions cell-mediated immune response and humoral immune response were highly enriched in goats at 5 dpv. At 14 dpv, cell-mediated immune response showed higher enrichment in goats, with humoral immune response being equally enriched. The antibody titer is reported to be lower in sheep compared to goats especially until 11 dpv (19). A plateau in antibody titer is maintained from 11 dpv onward, which is more evident in goats (19). So, it can be inferred that the magnitude of immune response was found to be higher and earlier in goats than in sheep.

## Conclusion

Proteome data analysis revealed that at the molecular level, the immune response produced by the PPRV vaccine virus is stronger in goat than that in sheep, but the vaccine provides equal protection in both. The altered expression of certain PBMC proteins especially ISG15 and IRF7 may result in marked changes in cellular signaling pathways to modulate host immune responses.

## Data Availability Statement

The raw data supporting the conclusions of this article will be made available by the authors, without undue reservation.

## Ethics Statement

The animal study was carried out after obtaining permission from Indian Veterinary Research Institute Animal Ethics Committee (IVRI - IAEC) under the Committee for Control and Supervision of Experiments on Animals(CPCSEA), India.

## Author Contributions

RS, BPM, and RG conceived and designed the research and proofread the manuscript. KR and DM performed the vaccine testing experiment. SW, ARS, and SS conducted the wet lab work. SW, ARS, RK, MP, and RG analyzed the data. SW, ARS, RK, AS, and BM helped in manuscript drafting and editing. All authors contributed to the article and approved the submitted version.

## Funding

This study was supported in part by the Centre for Agricultural Bioinformatics (ICAR-IASRI) (CABin/100644/16103/801/10133) and SubDIC (BTISnet), ICAR-IVRI.

## Conflict of Interest

The authors declare that the research was conducted in the absence of any commercial or financial relationships that could be construed as a potential conflict of interest.

## Publisher's Note

All claims expressed in this article are solely those of the authors and do not necessarily represent those of their affiliated organizations, or those of the publisher, the editors and the reviewers. Any product that may be evaluated in this article, or claim that may be made by its manufacturer, is not guaranteed or endorsed by the publisher.

## References

[1] Kamel M El-Sayed AJV. Toward peste des petits virus (PPRV) eradication: diagnostic approaches, novel vaccines, and control strategies. Virus Res. (2019) 274:197774. 10.1016/j.virusres.2019.197774 31606355

[2] Shaila MS Purushothaman V Bhavasar D Venugopal K Venkatesan RA. Peste des petits ruminants of sheep in India. Vet Rec. (1989) 125:602. 2609485

[3] Singh RK Balamurugan V Bhanuprakash V Sen A Saravanan P Pal Yadav M. Possible control and eradication of peste des petits ruminants from India: technical aspects. Vet Ital. (2009) 45:449–62. 20391409

[4] Diallo A Taylor WP Lefevre PC Provost A. Attenuation of a strain of rinderpest virus: potential homologous live vaccine. Revue d'elevage et de medecine veterinaire des pays tropicaux. Rev Elev Med Vet Pays Trop. (1989) 42:311–9. 2485537

[5] Saravanan P Sen A Balamurugan V Rajak KK Bhanuprakash V Palaniswami KS. Comparative efficacy of peste des petits ruminants (PPR) vaccines. Biologicals. (2010) 38:479–85. 10.1016/j.biologicals.2010.02.003 20199873

[6] Lund BT Tiwari A Galbraith S Baron MD Morrison WI Barrett T. Vaccination of cattle with attenuated rinderpest virus stimulates CD4(+) T cell responses with broad viral antigen specificity. J Gen Virol. (2000) 81:2137–46. 10.1099/0022-1317-81-9-2137 10950969

[7] Sinnathamby G Renukaradhya GJ Rajasekhar M Nayak R Shaila MS. Immune responses in goats to recombinant hemagglutinin-neuraminidase glycoprotein of Peste des petits ruminants virus: identification of a T cell determinant. Vaccine. (2001) 19:4816–23. 10.1016/S0264-410X(01)00210-9 11535334

[8] Kumar N Maherchandani S Kashyap SK Singh SV Sharma S Chaubey KK. Peste des petits ruminants virus infection of small ruminants: a comprehensive review. Viruses. (2014) 6:2287–327. 10.3390/v6062287 24915458 PMC4074929

[9] Manjunath S Kumar GR Mishra BP Mishra B Sahoo AP Joshi CG. Genomic analysis of host–Peste des petits ruminants vaccine viral transcriptome uncovers transcription factors modulating immune regulatory pathways. Vet Res. (2015) 46:15. 10.1186/s13567-015-0153-8 25827022 PMC4337102

[10] Manjunath S Mishra BP Mishra B Sahoo AP Tiwari AK Rajak KK. Comparative and temporal transcriptome analysis of peste des petits ruminants virus infected goat peripheral blood mononuclear cells. Virus Res. (2017) 229:28–40. 10.1016/j.virusres.2016.12.014 28017736

[11] Wani SA Praharaj MR Sahu AR Khan RIN Saxena S Rajak KK. Systems Biology behind immunoprotection of both Sheep and Goats after Sungri/96 PPRV vaccination. mSystems. (2021) 6:e00820–20. 10.1128/mSystems.00820-20 33785572 PMC8546983

[12] van der Pouw Kraan TC Kasperkovitz PV Verbeet N Verweij CL. Genomics in the immune system. Clin Immunol. (2004) 111:175–85. 10.1016/j.clim.2004.01.001 15137950

[13] Bolt G Berg K Moller M. Measles virus-induced modulation of host-cell gene expression. J Gen Virol. (2002) 83:1157–65. 10.1099/0022-1317-83-5-1157 11961271

[14] Iwasa T Suga S Qi L Komada Y. Apoptosis of human peripheral blood mononuclear cells by wild-type measles virus infection is induced by interaction of hemagglutinin protein and cellular receptor, SLAM via caspase-dependent pathway. Microbiol Immunol. (2010) 54:405–16. 10.1111/j.1348-0421.2010.00231.x 20618687

[15] Pandey A Sahu AR Wani SA Saxena S Kanchan S Sah V. Modulation of Host miRNAs transcriptome in lung and spleen of Peste des petits ruminants virus infected sheep and goats. Front Microbiol. (2017) 8:1146. 10.3389/fmicb.2017.01146 28694795 PMC5483481

[16] Khanduri A Sahu AR Wani SA Khan RIN Pandey A Saxena S. Dysregulated miRNAome and proteome of PPRV infected goat PBMCs reveal a coordinated immune response. Front Immunol. (2018) 9:2631. 10.3389/fimmu.2018.02631 30524425 PMC6262310

[17] Singh RP De UK Pandey KD. Virological and antigenic characterization of two Peste des Petits Ruminants (PPR) vaccine viruses of Indian origin. Comp Immunol Microbiol Infect Dis. (2010) 33:343–53. 10.1016/j.cimid.2008.12.003 19200598

[18] Raj GD Nachimuthu K Nainar AM. A simplified objective method for quantification of peste des petits ruminants virus or neutralizing antibody. J Virol Methods. (2000) 89:89–95. 10.1016/S0166-0934(00)00206-8 10996642

[19] Wani SA Sahu AR Saxena S Rajak KK Saminathan M Sahoo AP. Expression kinetics of *ISG15, IRF3, IFN gamma, IL10, IL2 and IL4* genes vis-à-vis virus shedding, tissue tropism and antibody dynamics in Peste des petits ruminants virus vaccinated, challenged, infected sheep and goats. Microb Pathog. (2018) 117:206–18. 10.1016/j.micpath.2018.02.027 29476787

[20] Zheng B Xu G Chen X Marinova E Han S. ICOSL-mediated signaling is essential for the survival and functional maturation of germinal center B cells through the classical NF-κB pathway (IRM10P. 611). J Immunol. (2015) 194 (1 Suppl.). 131–9. Available online at: https://www.jimmunol.org/content/194/1_Supplement/131.9

[21] Werner M Hobeika E Jumaa H. Role of PI3K in the generation and survival of B cells. Immunol Rev. (2010) 237:55–71. 10.1111/j.1600-065X.2010.00934.x 20727029

[22] Coombs KM. Quantitative proteomics of complex mixtures. Expert Rev Proteomics. (2011) 8:659–77. 10.1586/epr.11.55 21999835

[23] Neilson KA Ali NA Muralidharan S Mirzaei M Mariani M Assadourian G. Less label, more free: approaches in label-free quantitative mass spectrometry. Proteomics. (2011) 11:535–53. 10.1002/pmic.201000553 21243637

[24] Zheng J Sugrue RJ Tang K. Mass spectrometry based proteomic studies on viruses and hosts – A review. Anal Chim Acta. (2011) 702:149–59. 10.1016/j.aca.2011.06.045 21839192 PMC7094357

[25] Wani SA Sahu AR Khan RIN Pandey A Saxena S Hosamani N. Contrasting gene expression profiles of monocytes and lymphocytes from Peste-Des-Petits-Ruminants virus infected goats. Front Immunol. (2019) 10:1463. 10.3389/fimmu.2019.01463 31333643 PMC6624447

[26] Werneke SW Schilte C Rohatgi A Monte KJ Michault A Arenzana-Seisdedos F. ISG15 is critical in the control of Chikungunya virus infection independent of UbE1L mediated conjugation. PLoS Pathog. (2011) 7:e1002322. 10.1371/journal.ppat.1002322 22028657 PMC3197620

[27] Zhao C Collins MN Hsiang TY Krug RM. Interferon-induced ISG15 pathway: an ongoing virus–host battle. Trends Microbiol. (2013) 21:181–6. 10.1016/j.tim.2013.01.005 23414970 PMC3622817

[28] McGillivary G Jordan ZB Peeples ME Bakaletz LO. Replication of respiratory syncytial virus is inhibited by the host defense molecule viperin. J Innate Immun. (2013) 5:60–71. 10.1159/000342473 23018837 PMC3595061

[29] Schneider WM Chevillotte MD Rice CM. Interferon-stimulated genes: a complex web of host defenses. Annu Rev Immunol. (2014) 32:513–45. 10.1146/annurev-immunol-032713-120231 24555472 PMC4313732

[30] Helbig KJ Beard MR. The role of viperin in the innate antiviral response. J Mol Biol. (2014) 426:1210–9. 10.1016/j.jmb.2013.10.019 24157441

[31] Chan YK Gack MU. RIG-I-like receptor regulation in virus infection and immunity. Curr Opin Virol. (2015) 12:7–14. 10.1016/j.coviro.2015.01.004 25644461 PMC5076476

[32] McDonald JU Kaforou M Clare S Hale C Ivanova M Huntley D. A simple screening approach to prioritize genes for functional analysis identifies a role for interferon regulatory factor 7 in the control of respiratory syncytial virus disease. mSystems. (2016) 1:e00051–16. 10.1128/mSystems.00051-16 27822537 PMC5069771

[33] Hatesuer B Hoang HTT Riese P Trittel S Gerhauser I Elbahesh H. Deletion of IRF3 and IRF7 genes in mice results in altered interferon pathway activation and granulocyte-dominated inflammatory responses to influenza an infection. J Innate Immun. (2017) 9:145–61. 10.1159/000450705 27811478 PMC6738875

[34] Arts RJ Joosten LA van der Meer JW Netea MG. TREM-1: intracellular signaling pathways and interaction with pattern recognition receptors. J Leukoc Biol. (2013) 93:209–15. 10.1189/jlb.0312145 23108097

[35] Roe K Gibot S Verma S. Triggering receptor expressed on myeloid cells-1 (TREM-1): a new player in antiviral immunity? Front Microbiol. (2014). 5:627. 10.3389/fmicb.2014.00627 25505454 PMC4244588

[36] Hayden MS West AP Ghosh S. NF-κB and the immune response. Oncogene. (2006) 25:6758–80. 10.1038/sj.onc.1209943 17072327

[37] Denoeud J Moser M. Role of CD27/CD70 pathway of activation in immunity and tolerance. J Leukoc Biol. (2011) 89:195–203. 10.1189/jlb.0610351 20699361

[38] Stone EL Pepper M Katayama CD Kerdiles YM Lai CY Emslie E. ICOS coreceptor signaling inactivates the transcription factor FOXO1 to promote Tfh cell differentiation. Immunity. (2015) 42:239–51. 10.1016/j.immuni.2015.01.017 25692700 PMC4334393

[39] Wikenheiser DJ Stumhofer JS. ICOS co-stimulation: friend or foe? Front Immunol. (2016) 7:304. 10.3389/fimmu.2016.00304 27559335 PMC4979228

