# Supplementary material for: Proteome Modulation in Peripheral Blood Mononuclear Cells of Peste des Petits Ruminants Vaccinated Goats and Sheep
Source: Front Vet Sci. 2021 Sep 22;8:670968. doi: 10.3389/fvets.2021.670968 (PMC8493254; doi:10.3389/fvets.2021.670968)
Supplement: Supplementary file 1 [file Data_Sheet_1.doc]

**Supplementary Table 1**: Functional gene ontology of differentially expressed proteins 5 dpv vaccinated goats

| Categories | Diseases or Functions Annotation | p-Value | Predicted Activation State | Activation z-score | # Molecules |
| --- | --- | --- | --- | --- | --- |
| Cell-To-Cell Signaling and Interaction, Cellular Growth and Proliferation | stimulation of cells | 2.23E-13 | Increased | 3.623 | 25 |
| Post-Translational Modification | phosphorylation of protein | 7.16E-14 | Increased | 3.494 | 39 |
| Gene Expression | transcription | 1.66E-17 | Increased | 3.416 | 80 |
| Gene Expression | transcription of RNA | 9.46E-17 | Increased | 3.253 | 74 |
| Gene Expression | expression of RNA | 1.73E-18 | Increased | 3.228 | 85 |
| Cell-To-Cell Signaling and Interaction, Cellular Growth and Proliferation, Hematological System Development and Function | stimulation of leukocytes | 3.90E-14 | Increased | 3.202 | 20 |
| Cell-To-Cell Signaling and Interaction, Hematological System Development and Function, Immune Cell Trafficking | adhesion of mononuclear leukocytes | 1.18E-13 | Increased | 3.179 | 18 |
| Free Radical Scavenging | generation of reactive oxygen species | 2.62E-13 | Increased | 3.166 | 21 |
| Cell-To-Cell Signaling and Interaction | activation of cells | 2.81E-41 | Increased | 2.932 | 82 |
| Cell Signaling, Post-Translational Modification | tyrosine phosphorylation of protein | 1.23E-10 | Increased | 2.924 | 17 |
| Cellular Movement | chemotaxis | 1.49E-12 | Increased | 2.901 | 33 |
| Cellular Movement, Hematological System Development and Function, Immune Cell Trafficking | Lymphocyte migration | 5.64E-20 | Increased | 2.889 | 34 |
| Cellular Development, Hematological System Development and Function, Hematopoiesis, Lymphoid Tissue Structure and Development | maturation of leukocytes | 1.22E-16 | Increased | 2.855 | 25 |
| Cellular Movement, Hematological System Development and Function, Immune Cell Trafficking, Inflammatory Response | cellular infiltration of phagocytes | 2.10E-11 | Increased | 2.84 | 18 |
| Cellular Movement, Hematological System Development and Function, Immune Cell Trafficking | cell movement of antigen presenting cells | 1.14E-16 | Increased | 2.794 | 31 |
| Lipid Metabolism, Small Molecule Biochemistry | synthesis of eicosanoid | 5.03E-17 | Increased | 2.773 | 25 |
| Lymphoid Tissue Structure and Development, Tissue Morphology | quantity of lymphoid cells | 1.59E-31 | Increased | 2.761 | 62 |
| Hematological System Development and Function, Lymphoid Tissue Structure and Development, Tissue Morphology | quantity of lymphocytes | 1.14E-30 | Increased | 2.688 | 61 |
| Cellular Movement | invasion of cells | 1.30E-12 | Increased | 2.682 | 44 |
| Cell Death and Survival, Organismal Injury and Abnormalities, Renal and Urological Disease | cell death of kidney cells | 1.69E-12 | Increased | 2.676 | 26 |
| Tissue Morphology | quantity of cells | 3.99E-28 | Increased | 2.619 | 91 |
| Cellular Development, Hematological System Development and Function, Hematopoiesis, Lymphoid Tissue Structure and Development | maturation of phagocytes | 6.13E-14 | Increased | 2.613 | 18 |
| Connective Tissue Development and Function, Tissue Development | growth of connective tissue | 8.76E-20 | Increased | 2.61 | 46 |
| Lymphoid Tissue Structure and Development, Tissue Morphology | quantity of lymphatic system cells | 1.85E-31 | Increased | 2.574 | 64 |
| Cellular Movement, Hematological System Development and Function, Immune Cell Trafficking, Inflammatory Response | cell movement of macrophages | 2.16E-15 | Increased | 2.535 | 26 |
| Molecular Transport | transport of molecule | 6.31E-14 | Increased | 2.535 | 64 |
| Cell-mediated Immune Response, Cellular Movement, Hematological System Development and Function, Immune Cell Trafficking | T cell migration | 4.32E-17 | Increased | 2.531 | 27 |
| Cell Signaling, Molecular Transport, Vitamin and Mineral Metabolism | quantity of Ca2+ | 1.33E-12 | Increased | 2.518 | 28 |
| Cellular Movement, Hematological System Development and Function, Immune Cell Trafficking, Inflammatory Response | cell movement of phagocytes | 1.35E-22 | Increased | 2.507 | 46 |
| Cell-To-Cell Signaling and Interaction, Hematological System Development and Function | activation of blood cells | 1.04E-37 | Increased | 2.486 | 69 |
| Cell-To-Cell Signaling and Interaction, Hematological System Development and Function, Immune Cell Trafficking, Inflammatory Response | priming of leukocytes | 3.34E-11 | Increased | 2.481 | 12 |
| Inflammatory Response | cell-mediated response | 3.02E-18 | Increased | 2.48 | 25 |
| Lipid Metabolism, Small Molecule Biochemistry | synthesis of lipid | 1.54E-12 | Increased | 2.457 | 38 |
| Lipid Metabolism, Small Molecule Biochemistry | synthesis of prostaglandin | 1.51E-16 | Increased | 2.433 | 22 |
| Free Radical Scavenging | formation of reactive oxygen species | 1.62E-10 | Increased | 2.425 | 12 |
| Cell-To-Cell Signaling and Interaction, Hematological System Development and Function, Immune Cell Trafficking, Inflammatory Response | activation of leukocytes | 1.09E-37 | Increased | 2.411 | 67 |
| Cell-To-Cell Signaling and Interaction, Hematological System Development and Function, Immune Cell Trafficking, Inflammatory Response | activation of lymphocytes | 6.93E-31 | Increased | 2.405 | 50 |
| Hematological System Development and Function, Lymphoid Tissue Structure and Development, Tissue Morphology | quantity of T lymphocytes | 3.25E-26 | Increased | 2.402 | 49 |
| Hematological System Development and Function, Tissue Morphology | quantity of mononuclear leukocytes | 1.33E-30 | Increased | 2.385 | 62 |
| Cell Death and Survival | killing of mononuclear leukocytes | 8.73E-11 | Increased | 2.36 | 10 |
| Cell-mediated Immune Response, Cellular Movement, Hematological System Development and Function, Immune Cell Trafficking | cell movement of T lymphocytes | 2.05E-17 | Increased | 2.343 | 26 |
| Cellular Movement, Hematological System Development and Function, Immune Cell Trafficking, Inflammatory Response | cellular infiltration by macrophages | 1.75E-11 | Increased | 2.335 | 17 |
| Cell-To-Cell Signaling and Interaction, Cellular Growth and Proliferation | induction of cells | 1.97E-10 | Increased | 2.335 | 14 |
| Cell Death and Survival, Organismal Injury and Abnormalities, Renal and Urological Disease | necrosis of kidney | 7.71E-13 | Increased | 2.32 | 27 |
| Molecular Transport | quantity of metal ion | 3.61E-13 | Increased | 2.317 | 30 |
| Cell-To-Cell Signaling and Interaction, Cellular Movement, Hematological System Development and Function, Immune Cell Trafficking | recruitment of antigen presenting cells | 1.04E-11 | Increased | 2.317 | 15 |
| Cell-To-Cell Signaling and Interaction, Hematological System Development and Function, Immune Cell Trafficking, Inflammatory Response | activation of mononuclear leukocytes | 7.07E-32 | Increased | 2.292 | 52 |
| Free Radical Scavenging | synthesis of reactive oxygen species | 7.60E-23 | Increased | 2.288 | 43 |
| Cell-To-Cell Signaling and Interaction, Hematological System Development and Function, Immune Cell Trafficking, Inflammatory Response | activation of antigen presenting cells | 1.44E-22 | Increased | 2.282 | 35 |
| Cellular Development, Hematological System Development and Function, Hematopoiesis, Lymphoid Tissue Structure and Development | maturation of antigen presenting cells | 1.69E-12 | Increased | 2.263 | 16 |
| Cell-To-Cell Signaling and Interaction, Hematological System Development and Function, Immune Cell Trafficking | adhesion of lymphocytes | 9.74E-11 | Increased | 2.237 | 14 |
| Cell-To-Cell Signaling and Interaction, Hematological System Development and Function, Immune Cell Trafficking, Inflammatory Response | priming of mononuclear leukocytes | 7.90E-11 | Increased | 2.228 | 11 |
| Cellular Movement | cell movement of myeloid cells | 7.00E-22 | Increased | 2.219 | 45 |
| Cellular Development, Cellular Growth and Proliferation, Organ Development, Skeletal and Muscular System Development and Function, Tissue Development | proliferation of smooth muscle cells | 3.93E-14 | Increased | 2.217 | 25 |
| Cellular Growth and Proliferation, Connective Tissue Development and Function, Tissue Development | proliferation of connective tissue cells | 1.67E-16 | Increased | 2.212 | 40 |
| Cell-To-Cell Signaling and Interaction, Cell-mediated Immune Response, Cellular Movement, Hematological System Development and Function, Immune Cell Trafficking | adhesion of T lymphocytes | 6.30E-11 | Increased | 2.206 | 13 |
| Cellular Development | maturation of cells | 1.07E-13 | Increased | 2.188 | 31 |
| Cell-To-Cell Signaling and Interaction, Hematological System Development and Function, Immune Cell Trafficking, Inflammatory Response | activation of macrophages | 2.01E-20 | Increased | 2.184 | 29 |
| Gene Expression | induction of RNA | 7.04E-11 | Increased | 2.18 | 10 |
| Cell Death and Survival, Cellular Compromise | cytotoxicity of cells | 2.68E-18 | Increased | 2.159 | 26 |
| Cell Signaling, Small Molecule Biochemistry | synthesis of nitric oxide | 2.91E-21 | Increased | 2.149 | 32 |
| Cellular Movement, Hematological System Development and Function, Immune Cell Trafficking | cell movement of lymphocytes | 1.38E-22 | Increased | 2.147 | 39 |
| Cell-To-Cell Signaling and Interaction, Nervous System Development and Function | activation of neuroglia | 2.73E-13 | Increased | 2.134 | 17 |
| Cellular Development, Cellular Growth and Proliferation, Organ Development, Skeletal and Muscular System Development and Function, Tissue Development | proliferation of muscle cells | 1.68E-12 | Increased | 2.134 | 27 |
| Cellular Movement, Immune Cell Trafficking | leukocyte migration | 5.26E-36 | Increased | 2.125 | 73 |
| Hematological System Development and Function | hemostasis | 5.03E-14 | Increased | 2.12 | 22 |
| Cellular Development, Cellular Growth and Proliferation, Hematological System Development and Function, Lymphoid Tissue Structure and Development | cell proliferation of T lymphocytes | 3.68E-23 | Increased | 2.074 | 48 |
| Inflammatory Response | immune response of cells | 2.23E-30 | Increased | 2.042 | 55 |
| Cell-To-Cell Signaling and Interaction, Cellular Growth and Proliferation | induction of blood cells | 1.11E-11 | Increased | 2.04 | 13 |
| Cellular Movement, Hematological System Development and Function, Immune Cell Trafficking | cell movement of leukocytes | 4.10E-33 | Increased | 2.037 | 66 |
| Cellular Movement | cell movement | 1.55E-29 | Increased | 2.036 | 103 |
| DNA Replication, Recombination, and Repair | synthesis of DNA | 9.50E-12 | Increased | 2.025 | 27 |
| Cell-To-Cell Signaling and Interaction, Hematological System Development and Function, Immune Cell Trafficking | adhesion of immune cells | 5.96E-19 | Increased | 2.009 | 33 |
| Cell-To-Cell Signaling and Interaction | adhesion of blood cells | 1.06E-19 | Increased | 2.007 | 35 |
| Tissue Morphology | quantity of bacteria | 3.18E-12 | Decreased | -2.01 | 11 |
| Cancer, Organismal Injury and Abnormalities | abdominal cancer | 1.49E-13 | Decreased | -2.036 | 201 |
| Cancer, Gastrointestinal Disease, Hepatic System Disease, Organismal Injury and Abnormalities | liver tumor | 1.52E-10 | Decreased | -2.094 | 114 |
| Cancer, Organismal Injury and Abnormalities | epithelial cancer | 1.24E-15 | Decreased | -2.244 | 205 |
| Cancer, Gastrointestinal Disease, Hepatic System Disease, Organismal Injury and Abnormalities | hepatobiliary system cancer | 4.09E-11 | Decreased | -2.577 | 115 |
| Cancer, Gastrointestinal Disease, Hepatic System Disease, Organismal Injury and Abnormalities | liver cancer | 6.47E-11 | Decreased | -2.578 | 113 |
| Cancer, Gastrointestinal Disease, Hepatic System Disease, Organismal Injury and Abnormalities | liver carcinoma | 8.35E-11 | Decreased | -2.795 | 110 |
| Organismal Injury and Abnormalities | Bleeding | 5.92E-20 | Decreased | -3.734 | 38 |
| Organismal Survival | morbidity or mortality | 5.01E-32 | Decreased | -4.888 | 113 |
| Organismal Survival | organismal death | 7.42E-32 | Decreased | -5.087 | 112 |

**Supplementary Table 2:** Functional gene ontology of differentially expressed proteins 14dpv vaccinated goats

| Categories | Diseases or Functions Annotation | p-Value | Predicted Activation State | Activation z-score | # Molecules |
| --- | --- | --- | --- | --- | --- |
| Cell Death and Survival, Organismal Injury and Abnormalities | apoptosis of epithelial cells | 1.17E-33 | Increased | 3.145 | 61 |
| Cellular Function and Maintenance | function of mononuclear leukocytes | 1.72E-29 | Increased | 2.916 | 59 |
| Inflammatory Response | immune response of cells | 2.49E-55 | Increased | 2.837 | 114 |
| Cellular Function and Maintenance | function of lymphatic system cells | 7.15E-31 | Increased | 2.761 | 62 |
| Cell Death and Survival | cell death | 9.87E-76 | Increased | 2.695 | 320 |
| Cellular Function and Maintenance, Hematological System Development and Function | function of lymphocytes | 6.78E-28 | Increased | 2.599 | 57 |
| Humoral Immune Response, Protein Synthesis | production of antibody | 7.58E-25 | Increased | 2.585 | 54 |
| Cellular Function and Maintenance, Hematological System Development and Function | function of T lymphocytes | 7.84E-26 | Increased | 2.582 | 50 |
| Cellular Function and Maintenance | function of leukocytes | 3.84E-40 | Increased | 2.581 | 88 |
| Cell Death and Survival, Gastrointestinal Disease, Hepatic System Disease, Organismal Injury and Abnormalities | cell death of hepatocytes | 2.03E-24 | Increased | 2.574 | 38 |
| Cellular Function and Maintenance | function of blood cells | 3.25E-47 | Increased | 2.568 | 101 |
| Cell Death and Survival, Gastrointestinal Disease, Hepatic System Disease, Organismal Injury and Abnormalities | necrosis of liver | 2.71E-32 | Increased | 2.325 | 55 |
| Cell Death and Survival | necrosis | 4.84E-69 | Increased | 2.261 | 273 |
| Organismal Injury and Abnormalities | Bleeding | 4.62E-70 | Increased | 2.211 | 114 |
| Protein Synthesis | production of protein | 1.11E-28 | Increased | 2.198 | 60 |
| Gene Expression | transcription | 4.17E-48 | Increased | 2.187 | 205 |
| Cell-To-Cell Signaling and Interaction | response of antigen presenting cells | 2.31E-29 | Increased | 2.13 | 48 |
| Gene Expression | transcription of RNA | 3.00E-42 | Increased | 2.11 | 184 |
| Cell Death and Survival | cell viability of mononuclear leukocytes | 5.38E-25 | Increased | 2.088 | 43 |
| Skeletal and Muscular System Development and Function | function of muscle | 4.36E-31 | Increased | 2.034 | 63 |
| Cell Death and Survival, Organismal Injury and Abnormalities | necrosis of epithelial tissue | 9.70E-52 | Increased | 2.019 | 111 |
| Cell-To-Cell Signaling and Interaction, Hematological System Development and Function | response of lymphocytes | 6.64E-26 | Increased | 2.004 | 44 |
| Cardiovascular System Development and Function, Organismal Development | vasculogenesis | 3.71E-107 | Decreased | -2.006 | 184 |
| Cardiovascular System Development and Function | vasoconstriction | 9.60E-26 | Decreased | -2.015 | 34 |
| Cell Death and Survival | cell death of mononuclear leukocytes | 5.24E-31 | Decreased | -2.021 | 69 |
| Hematological System Development and Function | coagulation | 1.29E-60 | Decreased | -2.037 | 73 |
| Cardiovascular System Development and Function, Organismal Development | angiogenesis | 9.45E-131 | Decreased | -2.038 | 223 |
| Cancer, Cellular Development, Cellular Growth and Proliferation, Organismal Injury and Abnormalities, Tumor Morphology | proliferation of cancer cells | 1.60E-26 | Decreased | -2.048 | 61 |
| Cellular Movement | cell movement of muscle cells | 1.77E-26 | Decreased | -2.066 | 43 |
| Cardiovascular System Development and Function | development of vasculature | 1.68E-130 | Decreased | -2.1 | 235 |
| Cell Death and Survival | apoptosis of lymphocytes | 7.97E-31 | Decreased | -2.106 | 64 |
| Cellular Movement | cell movement of connective tissue cells | 1.13E-26 | Decreased | -2.129 | 49 |
| Cell Morphology, Tissue Development | tubulation of cells | 2.79E-25 | Decreased | -2.136 | 38 |
| Cardiovascular System Development and Function | vasoconstriction of blood vessel | 4.02E-24 | Decreased | -2.156 | 32 |
| Hematological System Development and Function, Tissue Morphology | quantity of granulocytes | 4.22E-30 | Decreased | -2.177 | 61 |
| Infectious Diseases | replication of RNA virus | 4.23E-31 | Decreased | -2.188 | 78 |
| Cellular Movement, Skeletal and Muscular System Development and Function | cell movement of smooth muscle cells | 1.08E-25 | Decreased | -2.204 | 40 |
| Cell Death and Survival | apoptosis of mononuclear leukocytes | 1.41E-31 | Decreased | -2.211 | 66 |
| Cancer, Cellular Development, Cellular Growth and Proliferation, Organismal Injury and Abnormalities, Tumor Morphology | proliferation of tumor cells | 4.58E-29 | Decreased | -2.224 | 73 |
| Infectious Diseases | Viral Infection | 6.46E-51 | Decreased | -2.26 | 186 |
| Infectious Diseases | replication of virus | 1.48E-31 | Decreased | -2.262 | 83 |
| Cell Death and Survival | apoptosis of lymphatic system cells | 8.48E-33 | Decreased | -2.299 | 69 |
| Cell Cycle | mitosis | 2.29E-25 | Decreased | -2.457 | 71 |
| Cell-To-Cell Signaling and Interaction | aggregation of cells | 9.35E-47 | Decreased | -2.537 | 80 |
| Hematological System Development and Function | hemostasis | 5.21E-62 | Decreased | -2.548 | 78 |
| Behavior | behavior | 4.08E-27 | Decreased | -2.59 | 111 |
| Cancer, Organismal Injury and Abnormalities | advanced malignant tumor | 3.24E-35 | Decreased | -3.052 | 119 |
| Cancer, Organismal Injury and Abnormalities | metastasis | 5.93E-34 | Decreased | -3.152 | 111 |

**Supplementary Table 3:** Functional gene ontology of differentially expressed proteins 5dpv vaccinated sheep

| Categories | Diseases or Functions Annotation | p-Value | Predicted Activation State | Activation z-score | # Molecules |
| --- | --- | --- | --- | --- | --- |
| Humoral Immune Response, Protein Synthesis | quantity of IgG | 2.82E-13 | Increased | 3.276 | 19 |
| Humoral Immune Response, Protein Synthesis | quantity of immunoglobulin | 6.27E-20 | Increased | 3.175 | 28 |
| Hematological System Development and Function, Hematopoiesis, Lymphoid Tissue Structure and Development, Organ Morphology, Tissue Morphology | quantity of double-positive thymocyte | 3.44E-08 | Increased | 3 | 10 |
| Protein Synthesis | production of protein | 5.07E-23 | Increased | 2.605 | 32 |
| Humoral Immune Response, Protein Synthesis | quantity of IgG1 | 7.04E-13 | Increased | 2.442 | 15 |
| Hematological System Development and Function, Tissue Development | accumulation of myeloid cells | 8.64E-10 | Increased | 2.228 | 14 |
| Cancer, Organismal Injury and Abnormalities | melanoma | 7.11E-09 | Increased | 2.213 | 118 |
| Hematological System Development and Function, Hematopoiesis, Lymphoid Tissue Structure and Development, Organ Morphology, Tissue Morphology | quantity of thymocytes | 4.18E-10 | Increased | 2.192 | 16 |
| Hematological System Development and Function, Inflammatory Response, Tissue Morphology | quantity of dendritic cells | 4.24E-12 | Increased | 2.04 | 14 |
| Gastrointestinal Disease, Inflammatory Response, Organismal Injury and Abnormalities | inflammation of intestine | 1.06E-18 | Decreased | -2.016 | 30 |
| Inflammatory Response | inflammation of absolute anatomical region | 2.02E-29 | Decreased | -2.02 | 64 |
| Cell Death and Survival | cell death of macrophages | 9.99E-10 | Decreased | -2.027 | 12 |
| Inflammatory Response, Organismal Injury and Abnormalities | inflammation of organ | 5.30E-30 | Decreased | -2.042 | 69 |
| Cellular Development | maturation of cells | 7.87E-14 | Decreased | -2.076 | 27 |
| Cell Death and Survival, Organismal Injury and Abnormalities | cell death of epithelial cells | 3.50E-08 | Decreased | -2.086 | 21 |
| Cellular Movement | cellular infiltration | 2.32E-20 | Decreased | -2.109 | 34 |
| Cell Death and Survival | cytotoxicity | 6.34E-22 | Decreased | -2.219 | 27 |
| Cell Signaling, Molecular Transport, Small Molecule Biochemistry | release of nitric oxide | 2.15E-08 | Decreased | -2.282 | 9 |
| Inflammatory Response | inflammation of body cavity | 4.09E-26 | Decreased | -2.362 | 56 |
| Organismal Survival | morbidity or mortality | 6.73E-17 | Decreased | -2.385 | 73 |
| Organismal Survival | organismal death | 1.28E-16 | Decreased | -2.469 | 72 |
| Cell Death and Survival, Organismal Injury and Abnormalities | necrosis of epithelial tissue | 4.25E-10 | Decreased | -2.743 | 26 |

**Supplementary Table 4:** Functional gene ontology of differentially expressed proteins 14dpv vaccinated sheep

| Categories | Diseases or Functions Annotation | p-Value | Predicted Activation State | Activation z-score | # Molecules |
| --- | --- | --- | --- | --- | --- |
| Hematological System Development and Function, Tissue Morphology | quantity of leukocytes | 2.67E-41 | Increased | 3.849 | 76 |
| Lymphoid Tissue Structure and Development, Tissue Morphology | quantity of lymphatic system cells | 1.77E-37 | Increased | 3.826 | 67 |
| Hematological System Development and Function, Tissue Morphology | quantity of mononuclear leukocytes | 1.68E-36 | Increased | 3.825 | 65 |
| Cellular Development, Cellular Growth and Proliferation, Hematological System Development and Function, Humoral Immune Response, Lymphoid Tissue Structure and Development | proliferation of B lymphocytes | 5.33E-24 | Increased | 3.745 | 34 |
| Lymphoid Tissue Structure and Development, Tissue Morphology | quantity of lymphoid cells | 1.78E-36 | Increased | 3.71 | 64 |
| Hematological System Development and Function, Lymphoid Tissue Structure and Development, Tissue Morphology | quantity of lymphocytes | 1.54E-35 | Increased | 3.586 | 63 |
| Cell-To-Cell Signaling and Interaction | activation of cells | 1.37E-48 | Increased | 3.4 | 85 |
| Hematological System Development and Function, Tissue Morphology | quantity of blood cells | 2.55E-41 | Increased | 3.329 | 80 |
| Cell-To-Cell Signaling and Interaction, Hematological System Development and Function | activation of blood cells | 6.81E-50 | Increased | 3.275 | 77 |
| Cell-To-Cell Signaling and Interaction, Hematological System Development and Function, Immune Cell Trafficking, Inflammatory Response | activation of natural killer cells | 2.10E-15 | Increased | 3.256 | 18 |
| Cellular Development, Cellular Growth and Proliferation, Hematological System Development and Function, Lymphoid Tissue Structure and Development | proliferation of mononuclear leukocytes | 4.10E-47 | Increased | 3.156 | 75 |
| Cell-To-Cell Signaling and Interaction, Hematological System Development and Function, Immune Cell Trafficking | adhesion of immune cells | 1.67E-23 | Increased | 3.131 | 36 |
| Cellular Development, Cellular Growth and Proliferation | proliferation of blood cells | 8.14E-47 | Increased | 3.102 | 79 |
| Cell-To-Cell Signaling and Interaction, Hematological System Development and Function, Immune Cell Trafficking, Inflammatory Response | priming of leukocytes | 9.72E-12 | Increased | 3.1 | 12 |
| Hematological System Development and Function, Humoral Immune Response, Lymphoid Tissue Structure and Development, Tissue Morphology | quantity of B lymphocytes | 1.95E-14 | Increased | 3.053 | 27 |
| Cell Death and Survival | cell viability of mononuclear leukocytes | 1.67E-25 | Increased | 3.032 | 30 |
| Cell-To-Cell Signaling and Interaction, Hematological System Development and Function, Immune Cell Trafficking, Inflammatory Response | activation of mononuclear leukocytes | 2.46E-42 | Increased | 3.009 | 59 |
| Cell-To-Cell Signaling and Interaction, Hematological System Development and Function, Immune Cell Trafficking, Inflammatory Response | activation of leukocytes | 1.32E-47 | Increased | 3.006 | 73 |
| Cellular Growth and Proliferation, Lymphoid Tissue Structure and Development | proliferation of lymphatic system cells | 4.78E-48 | Increased | 2.956 | 78 |
| Cell Death and Survival | cell viability of blood cells | 5.58E-29 | Increased | 2.947 | 38 |
| Cellular Development, Cellular Growth and Proliferation, Hematological System Development and Function, Lymphoid Tissue Structure and Development | proliferation of lymphocytes | 2.50E-45 | Increased | 2.937 | 73 |
| Hematological System Development and Function, Hematopoiesis, Lymphoid Tissue Structure and Development, Organ Morphology, Tissue Morphology | quantity of thymocytes | 1.37E-11 | Increased | 2.935 | 19 |
| Cell Death and Survival | cell viability of leukocytes | 7.06E-30 | Increased | 2.85 | 37 |
| Cell Death and Survival | cell viability of lymphatic system cells | 9.09E-24 | Increased | 2.831 | 29 |
| Cell-To-Cell Signaling and Interaction | priming of cells | 1.88E-12 | Increased | 2.777 | 13 |
| Hematological System Development and Function, Lymphoid Tissue Structure and Development, Tissue Morphology | quantity of natural killer cells | 5.65E-12 | Increased | 2.755 | 14 |
| Cell Death and Survival, Hematological System Development and Function | cell viability of lymphocytes | 1.35E-23 | Increased | 2.738 | 28 |
| Cellular Development | maturation of cells | 1.78E-18 | Increased | 2.714 | 35 |
| Cancer, Organismal Injury and Abnormalities | growth of malignant tumor | 1.07E-13 | Increased | 2.698 | 29 |
| Tissue Morphology | quantity of cells | 8.39E-31 | Increased | 2.694 | 89 |
| Cell-To-Cell Signaling and Interaction | adhesion of blood cells | 2.25E-25 | Increased | 2.664 | 39 |
| Cell-To-Cell Signaling and Interaction, Hematological System Development and Function, Immune Cell Trafficking, Inflammatory Response | activation of lymphocytes | 2.98E-41 | Increased | 2.641 | 57 |
| Cell-To-Cell Signaling and Interaction, Hematological System Development and Function, Immune Cell Trafficking | adhesion of mononuclear leukocytes | 2.67E-13 | Increased | 2.633 | 17 |
| Cellular Function and Maintenance | function of phagocytes | 5.46E-20 | Increased | 2.63 | 29 |
| Lymphoid Tissue Structure and Development, Organ Morphology, Tissue Morphology | quantity of thymus gland | 1.84E-12 | Increased | 2.628 | 20 |
| Cellular Development, Hematological System Development and Function, Hematopoiesis, Lymphoid Tissue Structure and Development | maturation of mononuclear leukocytes | 1.74E-11 | Increased | 2.592 | 15 |
| Cellular Development, Hematological System Development and Function, Hematopoiesis, Lymphoid Tissue Structure and Development | maturation of leukocytes | 1.91E-23 | Increased | 2.567 | 30 |
| Cellular Function and Maintenance | function of blood cells | 4.08E-38 | Increased | 2.541 | 58 |
| Cell-To-Cell Signaling and Interaction | activation of lymphatic system cells | 5.95E-42 | Increased | 2.504 | 58 |
| Cell-To-Cell Signaling and Interaction, Cellular Growth and Proliferation | stimulation of lymphatic system cells | 6.18E-15 | Increased | 2.5 | 18 |
| Cell-To-Cell Signaling and Interaction, Cellular Movement, Hematological System Development and Function, Immune Cell Trafficking | recruitment of granulocytes | 4.08E-17 | Increased | 2.493 | 24 |
| Cellular Function and Maintenance, Hematological System Development and Function | function of myeloid cells | 1.17E-13 | Increased | 2.433 | 20 |
| Cellular Development, Hematological System Development and Function, Hematopoiesis, Lymphoid Tissue Structure and Development | maturation of antigen presenting cells | 7.09E-17 | Increased | 2.418 | 19 |
| Humoral Immune Response, Protein Synthesis | quantity of IgG1 | 6.03E-14 | Increased | 2.399 | 17 |
| Cell-To-Cell Signaling and Interaction, Cellular Growth and Proliferation | stimulation of cells | 2.39E-16 | Increased | 2.394 | 27 |
| Hematological System Development and Function, Lymphoid Tissue Structure and Development, Tissue Morphology | quantity of T lymphocytes | 1.74E-30 | Increased | 2.369 | 51 |
| Cardiovascular System Development and Function, Cell-To-Cell Signaling and Interaction | adhesion of endothelial cells | 2.95E-13 | Increased | 2.36 | 17 |
| Cellular Development, Cellular Growth and Proliferation | proliferation of myeloid cells | 9.35E-12 | Increased | 2.346 | 17 |
| Cell-To-Cell Signaling and Interaction, Cellular Growth and Proliferation, Hematological System Development and Function | stimulation of lymphocytes | 2.40E-14 | Increased | 2.329 | 17 |
| Cellular Development, Hematological System Development and Function, Hematopoiesis | differentiation of hematopoietic progenitor cells | 2.74E-13 | Increased | 2.298 | 25 |
| Cellular Development, Cellular Growth and Proliferation, Hematological System Development and Function, Hematopoiesis, Lymphoid Tissue Structure and Development, Tissue Development | development of phagocytes | 7.56E-16 | Increased | 2.294 | 18 |
| Cellular Development, Cellular Growth and Proliferation, Hematological System Development and Function, Hematopoiesis | proliferation of hematopoietic progenitor cells | 7.32E-19 | Increased | 2.292 | 27 |
| Cell Death and Survival | cell viability | 8.49E-23 | Increased | 2.291 | 67 |
| Cell-To-Cell Signaling and Interaction, Cellular Growth and Proliferation, Hematological System Development and Function | stimulation of mononuclear leukocytes | 5.49E-16 | Increased | 2.291 | 19 |
| Cell Death and Survival | cell survival | 3.77E-24 | Increased | 2.272 | 71 |
| Humoral Immune Response, Protein Synthesis | production of antibody | 1.53E-26 | Increased | 2.268 | 37 |
| Cell-To-Cell Signaling and Interaction, Hematological System Development and Function, Immune Cell Trafficking, Inflammatory Response | activation of phagocytes | 8.78E-25 | Increased | 2.266 | 38 |
| Lymphoid Tissue Structure and Development, Tissue Morphology | quantity of lymphoid tissue | 3.03E-18 | Increased | 2.258 | 32 |
| Humoral Immune Response, Protein Synthesis | quantity of IgG | 7.50E-17 | Increased | 2.242 | 24 |
| Cellular Movement | migration of cells | 3.46E-35 | Increased | 2.241 | 98 |
| Cell Death and Survival | cell death of fibroblasts | 4.74E-12 | Increased | 2.218 | 21 |
| Cell-To-Cell Signaling and Interaction, Cellular Growth and Proliferation, Hematological System Development and Function | stimulation of leukocytes | 1.79E-18 | Increased | 2.214 | 23 |
| Hematological System Development and Function, Inflammatory Response, Tissue Morphology | quantity of macrophages | 7.85E-13 | Increased | 2.195 | 19 |
| Hematological System Development and Function, Tissue Morphology | quantity of antigen presenting cells | 5.99E-22 | Increased | 2.189 | 31 |
| Cell-To-Cell Signaling and Interaction, Hematological System Development and Function, Immune Cell Trafficking, Inflammatory Response | activation of antigen presenting cells | 4.55E-23 | Increased | 2.159 | 34 |
| Cell-To-Cell Signaling and Interaction, Cellular Movement, Hematological System Development and Function, Immune Cell Trafficking, Inflammatory Response | recruitment of neutrophils | 1.22E-17 | Increased | 2.126 | 23 |
| Cell-To-Cell Signaling and Interaction, Cellular Movement | recruitment of myeloid cells | 9.52E-23 | Increased | 2.121 | 32 |
| Cell-mediated Immune Response, Cellular Development, Cellular Function and Maintenance, Cellular Growth and Proliferation, Embryonic Development, Hematological System Development and Function, Hematopoiesis, Lymphoid Tissue Structure and Development, Organ Development, Organismal Development, Tissue Development | differentiation of helper T lymphocytes | 8.65E-22 | Increased | 2.12 | 27 |
| Cellular Development, Hematological System Development and Function, Hematopoiesis, Lymphoid Tissue Structure and Development | maturation of dendritic cells | 1.16E-14 | Increased | 2.109 | 17 |
| Cell-To-Cell Signaling and Interaction | response of antigen presenting cells | 6.44E-18 | Increased | 2.1 | 24 |
| Cell Death and Survival | cytolysis | 5.54E-14 | Increased | 2.092 | 23 |
| Hematological System Development and Function, Lymphoid Tissue Structure and Development, Tissue Morphology | quantity of CD8+ T lymphocyte | 1.69E-14 | Increased | 2.074 | 18 |
| Cancer, Organismal Injury and Abnormalities | sarcoma | 1.68E-11 | Increased | 2.066 | 38 |
| Cellular Function and Maintenance | function of leukocytes | 2.55E-36 | Increased | 2.064 | 54 |
| Cell Death and Survival | apoptosis of fibroblasts | 8.74E-12 | Increased | 2.054 | 19 |
| Cellular Function and Maintenance | engulfment of cells | 2.54E-12 | Increased | 2.042 | 27 |
| Cellular Development, Cellular Growth and Proliferation | expansion of blood cells | 1.95E-30 | Increased | 2.039 | 36 |
| Inflammatory Response | immune response of cells | 1.75E-40 | Increased | 2.032 | 62 |
| Connective Tissue Development and Function, Tissue Development | growth of connective tissue | 6.47E-21 | Increased | 2.023 | 45 |
| Cell Death and Survival, Hematological System Development and Function | cell viability of B lymphocytes | 7.72E-23 | Increased | 2.015 | 22 |
| Cell Death and Survival | apoptosis of mononuclear leukocytes | 1.02E-29 | Decreased | -2.137 | 42 |
| Cell Death and Survival | cell death of lymphocytes | 3.56E-27 | Decreased | -2.199 | 41 |
| Cell Death and Survival | apoptosis of lymphocytes | 4.05E-28 | Decreased | -2.456 | 40 |
| Infectious Diseases | replication of Herpesviridae | 2.15E-21 | Decreased | -2.926 | 18 |

**Supplementary Table 5: Networks generated in vaccinated Goats at 5dpv**

| **ID** | **Score** | **Focus Molecules** | **Top Diseases and Functions** |
| --- | --- | --- | --- |
| 1 | 43 | 24 | Infectious Diseases, Antimicrobial Response, Inflammatory Response |
| 2 | 36 | 21 | Lipid Metabolism, Molecular Transport, Small Molecule Biochemistry |
| 3 | 31 | 19 | Antimicrobial Response, Inflammatory Response, Infectious Diseases |
| 4 | 27 | 17 | Humoral Immune Response, Protein Synthesis, Embryonic Development |
| 5 | 25 | 16 | Cell-To-Cell Signaling and Interaction, Hematological System Development and Function, Immune Cell Trafficking |
| 6 | 25 | 16 | Inflammatory Response, Immunological Disease, Cell-To-Cell Signaling and Interaction |
| 7 | 21 | 14 | Cellular Movement, Hematological System Development and Function, Immune Cell Trafficking |
| 8 | 19 | 13 | Hematological System Development and Function, Organismal Functions, Hematological Disease |
| 9 | 15 | 11 | Cell Signaling, Cellular Function and Maintenance, Molecular Transport |
| 10 | 15 | 11 | Cell Death and Survival, Lipid Metabolism, Molecular Transport |
| 11 | 13 | 10 | Cellular Movement, Immune Cell Trafficking, Hematological System Development and Function |
| 12 | 13 | 10 | Organismal Functions, Cardiovascular Disease, Cell Death and Survival |
| 13 | 13 | 10 | Cellular Development, Hematological System Development and Function, Hematopoiesis |
| 14 | 11 | 9 | Cardiovascular System Development and Function, Connective Tissue Disorders, Dermatological Diseases and Conditions |
| 15 | 8 | 7 | Amino Acid Metabolism, Hematological Disease, Metabolic Disease |
| 16 | 7 | 6 | Antimicrobial Response, Inflammatory Response, Cell Morphology |
| 17 | 7 | 6 | Cardiovascular Disease, Cardiovascular System Development and Function, Developmental Disorder |
| 18 | 2 | 1 | Cell Cycle, Cell Morphology, Cellular Compromise |
| 19 | 2 | 1 | Connective Tissue Disorders, Developmental Disorder, Gastrointestinal Disease |

**Supplementary Table 6: Networks generated in vaccinated goats at 14dpv**

| **ID** | **Score** | **Focus Molecules** | **Top Diseases and Functions** |
| --- | --- | --- | --- |
| 1 | 40 | 27 | Developmental Disorder, Hereditary Disorder, Metabolic Disease |
| 2 | 36 | 25 | Antimicrobial Response, Inflammatory Response, Infectious Diseases |
| 3 | 33 | 24 | Cardiovascular System Development and Function, Organismal Development, Post-Translational Modification |
| 4 | 27 | 21 | Cardiovascular System Development and Function, Organismal Development, Cardiovascular Disease |
| 5 | 27 | 21 | Cell Cycle, Hematological System Development and Function, Tissue Morphology |
| 6 | 27 | 21 | Embryonic Development, Organ Development, Organismal Development |
| 7 | 26 | 20 | Lipid Metabolism, Molecular Transport, Small Molecule Biochemistry |
| 8 | 24 | 19 | Molecular Transport, Organismal Injury and Abnormalities, Cardiovascular System Development and Function |
| 9 | 24 | 19 | Cardiovascular System Development and Function, Hematological System Development and Function, Cellular Function and Maintenance |
| 10 | 24 | 19 | Cardiovascular System Development and Function, Hematological System Development and Function, Molecular Transport |
| 11 | 22 | 18 | Cardiovascular System Development and Function, Organismal Development, Cardiovascular Disease |
| 12 | 22 | 18 | Infectious Diseases, Cell-mediated Immune Response, Cellular Development |
| 13 | 22 | 18 | Cellular Movement, Hematological System Development and Function, Immune Cell Trafficking |
| 14 | 20 | 17 | Cardiovascular System Development and Function, Organismal Development, Cardiovascular Disease |
| 15 | 20 | 17 | Cellular Development, Cellular Growth and Proliferation, Embryonic Development |
| 16 | 20 | 17 | Hematological System Development and Function, Organismal Functions, Infectious Diseases |
| 17 | 20 | 17 | Cellular Development, Cellular Growth and Proliferation, Hematological System Development and Function |
| 18 | 20 | 17 | Cell Morphology, Cellular Function and Maintenance, Free Radical Scavenging |
| 19 | 18 | 16 | Skeletal and Muscular Disorders, Skeletal and Muscular System Development and Function, Cancer |
| 20 | 18 | 16 | Cardiovascular System Development and Function, Organismal Development, Cell Death and Survival |
| 21 | 17 | 15 | Cardiovascular System Development and Function, Organismal Development, Cell-To-Cell Signaling and Interaction |
| 22 | 15 | 14 | Cell Signaling, Small Molecule Biochemistry, Cellular Development |
| 23 | 15 | 14 | Neurological Disease, Organismal Injury and Abnormalities, Gene Expression |
| 24 | 14 | 13 | Infectious Diseases, Antimicrobial Response, Inflammatory Response |
| 25 | 14 | 13 | Cardiovascular System Development and Function, Organismal Development, Cellular Movement |

**Supplementary Table 7: Networks generated in vaccinated sheep at 5dpv**

| **ID** | **Score** | **Focus Molecules** | **Top Diseases and Functions** |
| --- | --- | --- | --- |
| 1 | 36 | 20 | Antimicrobial Response, Inflammatory Response, Infectious Diseases |
| 2 | 34 | 19 | Antimicrobial Response, Inflammatory Response, Infectious Diseases |
| 3 | 29 | 17 | Cellular Function and Maintenance, Cellular Development, Cellular Growth and Proliferation |
| 4 | 27 | 16 | Humoral Immune Response, Protein Synthesis, Developmental Disorder |
| 5 | 25 | 15 | Cellular Development, Cellular Growth and Proliferation, Hematological System Development and Function |
| 6 | 22 | 14 | Cellular Movement, Immune Cell Trafficking, Cellular Development |
| 7 | 18 | 12 | Cellular Movement, Cell-To-Cell Signaling and Interaction, Cardiovascular Disease |
| 8 | 18 | 12 | Cell Death and Survival, Gene Expression, Cell-mediated Immune Response |
| 9 | 13 | 9 | Cancer, Connective Tissue Disorders, Organismal Injury and Abnormalities |
| 10 | 13 | 9 | Endocrine System Disorders, Gastrointestinal Disease, Inflammatory Disease |
| 11 | 9 | 7 | Cellular Movement, Hematological System Development and Function, Immune Cell Trafficking |
| 12 | 9 | 7 | Cellular Development, Cell Death and Survival, Skeletal and Muscular System Development and Function |
| 13 | 7 | 6 | Skeletal and Muscular System Development and Function, Cardiovascular System Development and Function, Organ Morphology |
| 14 | 1 | 1 | Carbohydrate Metabolism, Lipid Metabolism, Small Molecule Biochemistry |

**Supplementary Table 8: Networks generated in vaccinated Sheep at 14dpv**

| **ID** | **Score** | **Focus Molecules** | **Top Diseases and Functions** |
| --- | --- | --- | --- |
| 1 | 37 | 21 | Antimicrobial Response, Inflammatory Response, Infectious Diseases |
| 2 | 30 | 18 | Antimicrobial Response, Inflammatory Response, Infectious Diseases |
| 3 | 28 | 17 | Inflammatory Response, Organismal Injury and Abnormalities, Cellular Function and Maintenance |
| 4 | 25 | 16 | Developmental Disorder, Hereditary Disorder, Immunological Disease |
| 5 | 19 | 13 | Infectious Diseases, Antimicrobial Response, Inflammatory Response |
| 6 | 17 | 12 | Cancer, Organismal Injury and Abnormalities, Connective Tissue Disorders |
| 7 | 17 | 12 | Cell Morphology, Cellular Function and Maintenance, Lipid Metabolism |
| 8 | 17 | 12 | Lymphoid Tissue Structure and Development, Tissue Morphology, Hematological System Development and Function |
| 9 | 17 | 12 | Cellular Movement, Dermatological Diseases and Conditions, Organismal Injury and Abnormalities |
| 10 | 15 | 11 | Organismal Development, Organ Morphology, Reproductive System Development and Function |
| 11 | 14 | 10 | Cardiovascular System Development and Function, Cellular Development, Cellular Function and Maintenance |
| 12 | 12 | 9 | Cell Signaling, Cellular Function and Maintenance, Molecular Transport |
| 13 | 12 | 9 | Cell Death and Survival, Cancer, Cell-To-Cell Signaling and Interaction |
| 14 | 12 | 9 | Cancer, Endocrine System Disorders, Organismal Injury and Abnormalities |
| 15 | 8 | 7 | Cardiovascular System Development and Function, Organismal Development, Organismal Injury and Abnormalities |
| 16 | 7 | 6 | Cellular Development, Cellular Growth and Proliferation, Hematological System Development and Function |
| 17 | 4 | 4 | Cell Death and Survival, Cardiovascular System Development and Function, Embryonic Development |
| 18 | 1 | 1 | Dermatological Diseases and Conditions, Organismal Injury and Abnormalities, Cellular Movement |
